# The protease calpain2a limits innate immunity by targeting TRAF6 in teleost fish

**DOI:** 10.1038/s42003-023-04711-7

**Published:** 2023-03-31

**Authors:** Yang Chen, Pengfei Wang, Qi Li, Xiaolong Yan, Tianjun Xu

**Affiliations:** 1 Laboratory of Fish Molecular Immunology, College of Fisheries and Life Science, Shanghai Ocean University, Shanghai, China; 2 Laboratory of Marine Biology and Biotechnology, Qingdao National Laboratory for Marine Science and Technology, Qingdao, China

**Keywords:** Innate immunity, Immune evasion

## Abstract

TNF receptor-associated factor 6 (TRAF6) plays a key signal transduction role in both antibacterial and antiviral signaling pathways. However, the regulatory mechanisms of TRAF6 in lower vertebrates are less reported. In this study, we identify calpain2a, is a member of the calcium-dependent proteases family with unique hydrolytic enzyme activity, functions as a key regulator for antibacterial and antiviral immunity in teleost fish. Upon lipopolysaccharide (LPS) stimulation, knockdown of calpain2a promotes the upregulation of inflammatory cytokines. Mechanistically, calpain2a interacts with TRAF6 and reduces the protein level of TRAF6 by hydrolyzing. After loss of enzymatic activity, mutant calpain2a competitively inhibits dimer formation and auto-ubiquitination of TRAF6. Knockdown of calpain2a also promotes cellular antiviral response. Mutant calpain2a lacking hydrolase activity represses ubiquitination of IFN regulatory factor (IRF) 3/7 from TRAF6. Taken together, these findings classify calpain2a is a negative regulator of innate immune responses by targeting TRAF6 in teleost fish.

## Abstract (web summary)

Calpain2a, a calcium-dependent protease, acts as a negative regulator of antibacterial and antiviral immunity in teleost fish by interacting with TRAF6 to promote TRAF6 degradation and inhibit TRAF6 dimerization and auto-ubiquitination.

## Introduction

As the first line of defense against invading pathogens, innate immunity exerts an important role to protect the body from bacterial invasion and viral infections. Pattern-recognition receptors (PRRs) on the membrane immediately recognize pathogen-associated molecular patterns (PAMPs)^1–3^. The three most widely studied PRRs in innate immunity are Toll-like receptors (TLRs), RIG-I-like receptors (RLRs), NOD-like receptors (NLRs), which recognize different PAMPs and trigger signal cascades to activate proinflammatory cytokines and antiviral factor^4–6^.

TNF receptor associated factor (TRAF) 6 is an indispensable part of the fight against bacterial invasion and viral infections. As an important signal pathway activated by lipopolysaccharide (LPS), nuclear transcription factor-κB (NF-κB) pathway has been mapped out in detail. Most of protein functions in NF-κB signaling pathway has been extensively studied^1,7^. Myeloid differentiation factor 88 (MyD88) is first activated by TLRs in response to LPS, and IRAK4 acts as a mediator by activating IRAK1 and IRAK2, which link MyD88 to TRAF6^8–11^. Ubc13 and Uev1A catalyze the K63-linked ubiquitination of TRAF6, which transfers K63 ubiquitin chain to TAB2 and TAB3, leading to the activation of TAK1^12–16^. TRAF6-regulated IKK activator 2 (TRIKA2) which is composed of TAK1, TAB1 and TAB2, activates inhibitor of IκB kinase (IKK) α/β phosphorylation^17–19^. Transcription factor NF-κB enters the nucleus and promotes the production of inflammatory cytokines^20^. When TLR7/9 recognize the virus, MyD88-IRAKs-TRAF6 complex transmits signals to IFN regulatory factor (IRF) 7, promotes IRF7 ubiquitination and activates its phosphorylation into the nucleus^21,22^. In RLR signaling pathway, RIG-I and melanoma differentiation-associated gene 5 (MDA5) activate mitochondrial antiviral signaling protein (MAVS)^23,24^. The aggregation of MAVS is an important feature of the beginning of RLR signal transmission^23,25,26^. TRAF2/3/6 are the first factors activated by MAVS^27,28^. TRAF3 will activate TBK1-IRF3 signaling, and MAVS-TRAF6 complex will activate NF-κB signaling, which promotes IFN-1 expression^27,29^.

The molecular mechanism of TRAF6 activation has already been demonstrated. TRAF6 in dimer form undergoes auto-ubiquitination under the catalysis of Ubc13 and Uev1A, which is related to Ring finger domain and ZF fingers domain. The model of TRAF6/Ubc13~UB confirms the important role of TRAF6 dimer in the aggregation and transmission of K63 ubiquitin chain^30^. In addition, it has been reported that Ring finger and ZF fingers domain recruit E2~ub conjugates in the zebrafish TRAF6 crystal model, and it was discovered that TRAF6 could form a heterodimer with TRAF5 from the same TRAF family for the first time^16^. The deubiquitination family proteins: A20, CYLD, USP2a, USP4, and USP20 target TRAF6 to remove K63-linked ubiquitination and reduce downstream signaling^31–35^. The yeast two-hybrid system screen out the interaction between UBE2O and TRAF6, and confirm that this interaction directly affects the activation of TRAF6 by MyD88^36^. In the downstream signal transduction process of TRAF6, ECSIT as an intermediate protein interacts with TRAF6 and TAK1 respectively, and this interaction strengthens the binding of TRAF6 and TAK1^37^. In LPS-induced TLR4 signaling, PRDXs prevents TRAF6 from recruiting ECSIT to deliver K63 ubiquitin chain and also inhibits BECN1 activated by TRAF6 in autophagy pathway^38^. MST4 activates TRAF6 phosphorylation at Thr463 and Thr486, and inhibits TRAF6 dimerization and ubiquitination^39^. In mammals, the protein regulation mechanism of TRAF6 has been widely studied in antibacterial and antiviral immunity. However, the research of TRAF6 in lower vertebrates is less studied. The immune response in teleost fish mainly rely on TLR and RLR pathways to execute the immune response after pathogen infection. Therefore, it is crucial to explore the regulatory mechanism of TRAF6-mediated signaling pathways.

calpain2 (m-calpain) is a family of calcium-dependent proteases, which is composed of more than 16 members in mammals and many other organisms^40^. The earliest study of calpain protein family is related to cell movement^41^. calpain promotes cell migration, and was found to inhibit neutrophil migration and the activation of p38 MAPK and ERK MAPK^42^. The activity of hydrolase is one of important functions of calpain. In immune regulation, calpain-2 promotes the release of NF-κB by hydrolyzing IkBα and activates the signaling pathway^43^.

In this study, we have determined that calpain2a interacts with TRAF6 and acts as a negative regulator in antibacterial and antiviral responses in teleost fish, miiuy croaker (*Miichthys miiuy*). calpain2a promotes the degradation of TRAF6 protein level through its hydrolase activity. Meanwhile, mutant calpain2a lacking hydrolase activity inhibits the formation of K63 ubiquitin chain by inhibiting the dimer of TRAF6. It is reflected that both calpain2a and mutant calpain2a terminate the ubiquitination of TRAF6 to ECSIT/ BENC1/ IRF3/ IRF7. calpain2a stops NF-κB and IFN signaling process by targeting TRAF6. Our results provide a molecule for the study of TRAF6-mediated immune regulation in lower vertebrates.

## Results

### calpain2a interacts with TRAF6 and negatively regulates NF-κB signaling

**Fig. 1:**
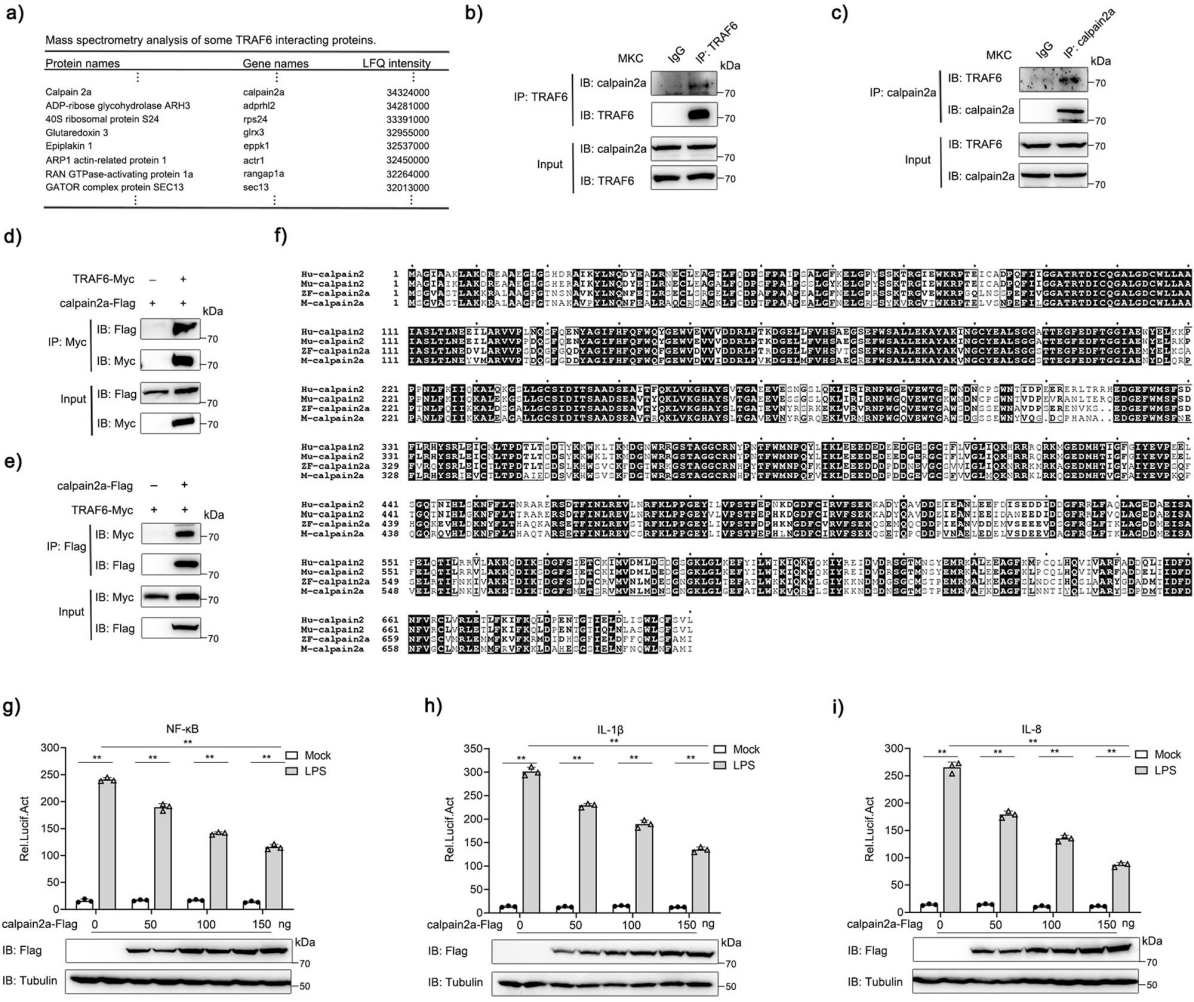
calpain2a interacts with TRAF6 and inhibits the NF-κB Signaling Pathway. **a** List of TRAF6 interactome based on Label-free quantification intensity (section). **b**, **c** MKC cells seeded in 6 cm^2^ dishes. After 24 h, cell lysates were immunoprecipitated (IP) with anti-TRAF6 or anti-calpain2a affinity gels. Then the immunoprecipitates and cell lysates were analyzed by IB with the anti-TRAF6 and anti-calpain2a Abs, respectively. **d**, **e** HEK 293 cells seeded in 6 cm^2^ dishes were transfected with the plasmids calpain2a-Flag and TRAF6-Myc (2 μg each). After 24 h, cell lysates were immunoprecipitated (IP) with anti-Myc **d** or anti-Flag **e** affinity gels. Then the immunoprecipitates and cell lysates were analyzed by IB with the anti-Myc and anti-Flag Abs, respectively. **f** The same amino acids among human calpain2a (Hu-calpain2), mouse calpain2a (Mu-calpain2), zebrafish calpain2a (ZF-calpain2a) and miiuy croaker calpain2a (M-calpain2a) are highlighted with black background. **g**–**i** EPC cells were transfected with calpain2a-Flag or empty vector together with the NF-κB, IL-1β, and IL-8 luciferase reporters. At 24 h post-transfection, cells were untreated (Mock) or treated with LPS for 6 h. The luciferase activity value was achieved against the Renilla luciferase activity (*n* = 3 per group). Western blot analysis was used to measure the expression of transiently transfected calpain2a-Flag. The expression of Tubulin was used as a loading control. Data were analyzed by two-way ANOVA (**g**, **h**, **i**). ^**^p  <  0.01. All experiments were performed in at least three independent experiments.

To gain insights into TRAF6-associated proteins in innate immunity response in teleost fish, we characterized the miiuy croaker TRAF6 interactome using affinity purification and mass spectrometry. Sorted by label-free quantification (LFQ) intensity, we intercepted part of the data and displayed it (Fig. 1a). Among TRAF6-associated proteins, calpain2a maybe regulated TRAF6 activity and affected TRAF6-mediated signaling pathways. Firstly, the relationship between calpain2a and TRAF6 at the protein level should be investigated, we checked the interaction of calpain2a with TRAF6 in Miiuy croaker kidney cell lines (MKC). Endogenous coimmunoprecipitation experiments indicated that calpain2a interacts with TRAF6 (Fig. 1b, c). As shown by transient transfection and coimmunoprecipitation experiments, overexpressed calpain2a and TRAF6 interacted with each other (Fig. 1d, e). All of these indicate calpain2a interacts with TRAF6 and further experiments are needed to investigate the mechanism of their action.

By Multiple sequence alignment of human, mouse, zebrafish, and miiuy croaker calpain2a proteins, calpain2a is evolutionarily conserved between fish and mammal (Fig. 1f). To determine the function of calpain2a in innate immunity, we examined whether calpain2a was associated with activation of NF-κB induced by LPS stimulation. We transfected epithelioma papulosum cyprinid (EPC) cells with NF-κB luciferase reporter, internal control renilla luciferase reporter, and vector encoding calpain2a or empty vector. calpain2a substantially reduced NF-κB reporter activity upon LPS stimulation in a dose-dependent manner (Fig. 1g). Moreover, proinflammatory cytokines such as IL-1β and IL-8 promoter responsive to LPS stimulation were markedly decreased in calpain2a-overexpressed EPC cells (Fig. 1h, i), suggesting a potential role for calpain2a in inflammatory signaling pathways induced by LPS stimulation.

### Negative regulation of LPS-induced NF-κB signaling by calpain2a

**Fig. 2:**
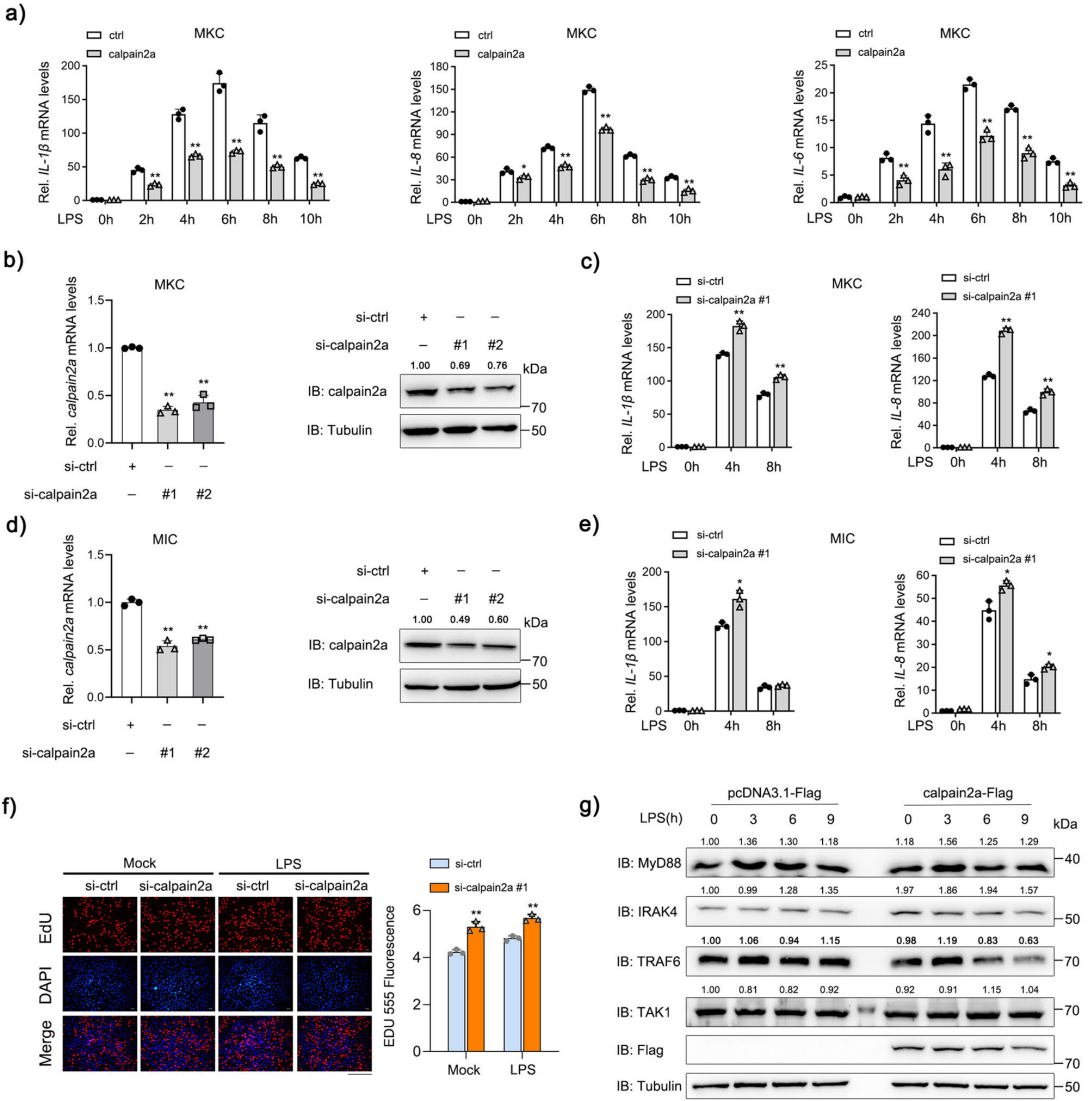
calpain2a negative regulates LPS-induced NF-κB Signaling Pathway. **a** *IL-1β*, *IL-8*, and *IL-6* mRNA in MKC transduced with calpain2a-Flag or empty vector and treated with saline (0) or challenged with LPS for various times (2 h, 4 h, 6 h, 8 h, 10 h) (*n* = 3 per group). **b**, **d** MKC cells and MIC cells were transfected with si-calpain2a #1 and si-calpain2a #2 (100 nM) or si-ctrl (100 nM). At 48 h post-transfection, *calpain2a* expression was measured by qRT-PCR (*n* = 3 per group). The level of calpain2a protein was determined by Western blotting. **c**, **e** *IL-1β*, *IL-8* mRNA in MKC and MIC stably transduced with si-calpain2a #1 or si-ctrl (control vector) and treated with saline (0) or challenged with LPS for various times (4 h, 8 h) (*n* = 3 per group). **f** MKC cells were transfected with either si-ctrl or si-calpain2a #1. At 36 h post-transfection, the cells were stimulated with LPS for 12 h, then cell proliferation assay was measured (*n* = 3 per group). Scale bar, 100 μm. **g** calpain2a-Flag was transfected into MKC cells which challenged with LPS for various times (3 h, 6 h, 9 h). Immunoblot analysis of cell lysates with indicated Abs. Relative mRNA level was normalized to the expression of the gene encoding *β-actin* in each sample. All experiments were performed in at least three independent experiments. Data were analyzed by two-way ANOVA (**a**, **c**, **e**, **f**) or one-way ANOVA (**b**, **d**). ^*^p  <  0.05, ^**^p  <  0.01.

To investigate the potential role of calpain2a in NF-κB signaling, we transfected calpain2a in MKC with a time gradient of LPS and assessed proinflammatory cytokines responsive to LPS. Overexpression of calpain2a led to downregulation of the expression of *IL-1β*, *IL-8*, and *IL-6* (Fig. 2a). Next, we designed two calpain2a-specific small interfering RNAs (siRNAs) efficiently downregulated calpain2a (Fig. 2b, d). Knockdown of calpain2a significantly enhanced LPS-induced proinflammatory cytokines including *IL-1β* and *IL-8* in MKC and Miiuy croaker intestine cell lines (MIC) (Fig. 2c, e). In the group of LPS-stimulated cells, knockdown of calpain2a enhanced cell proliferation. Similarly, in the absence of any stimulation, we found that knockdown of calpain2a also enhanced cell proliferation (Fig. 2f). To determine whether calpain2a inhibited proteins in LPS-induced NF-κB signaling, calpain2a markedly inhibited endogenous protein level of TRAF6 but not MyD88, IRAK4, or TAK1 (Fig. 2g). In summary, the kinetics of calpain2a and proinflammatory cytokine expression suggested functional involvement of calpain2a in LPS-induced NF-κB signaling.

### The interaction of calpain2a with TRAF6

**Fig. 3:**
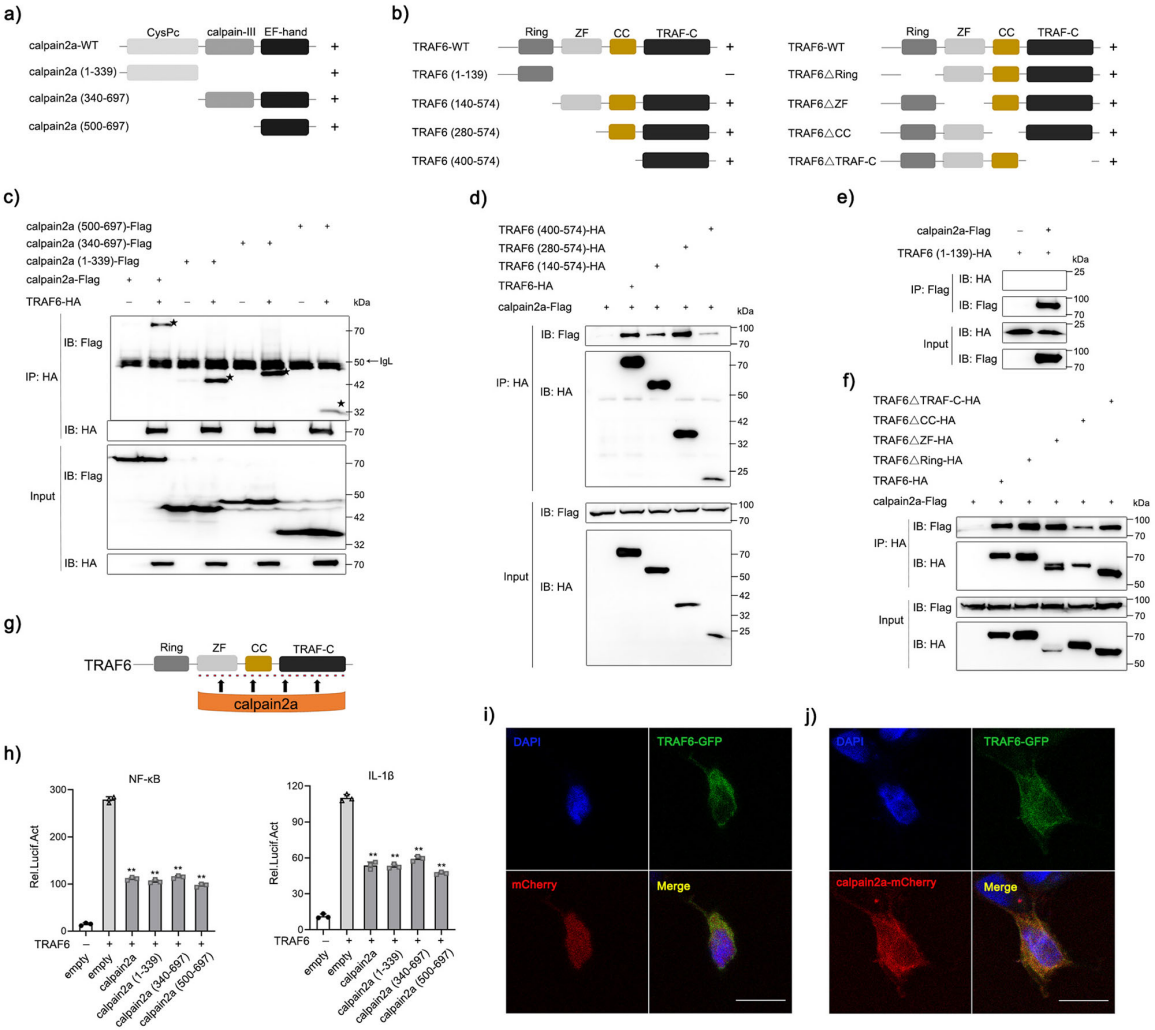
The interaction of calpain2a with TRAF6. **a**, **b** Schematic diagrams of domain organization in miiuy croaker calpain2a, TRAF6 and mutants used in this study. “+“ represents the interaction with TRAF6 or calpain2a, “-“ means no interaction. **c** HEK293 cells were transfected with mock, calpain2a-Flag wild type (WT), and calpain2a-Flag truncated mutants, or TRAF6-HA as indicated. At 24 h post-transfection, transfected cells were extracted and cell lysates were subjected to immunoprecipitation with anti-HA antibody followed by IB using anti-HA or anti-Flag antibody. **d**–**f** HEK293 cells were transfected with mock, TRAF6-HA wild type (WT), and TRAF6-HA truncated mutants, or calpain2a-Flag as indicated. At 24 h post-transfection, transfected cells were extracted and cell lysates were subjected to immunoprecipitation with anti-HA antibody **d**, **f** or anti-Flag antibody **e** followed by IB using anti-HA or anti-Flag antibody. **g** The interaction site of TRAF6 and calpain2a. **h** EPC cells were transfected with TRAF6-HA, calpain2a-Flag or calpain2a-Flag truncated mutants together with NF-κB and IL-1β luciferase reporters. After 36 h post-transfection, the luciferase activity value was achieved against the renilla luciferase activity (*n* = 3 per group). **i**, **j** EPC cells were transfected with mock, calpain2a-mCherry, and TRAF6-GFP. DAPI-stained nuclei are shown in blue. calpain2a was detected with red fluorescence, and TRAF6 was detected with green fluorescence. Scale bar, 10 μm. All experiments were performed in at least three independent experiments. Data were analyzed by one-way ANOVA (**h**). ^**^p  <  0.01.

Domain analysis was done using the Conserved Domain Database (CDD) NCBI. calpain2a comprises three domains: a Calpain family cysteine protease (CysPc) domain (aa 46-339), a central Calpain large subunit domain III (aa 360-499), and a C-terminal Penta-EF hand domain (aa 500-697). To determine which domain(s) of calpain2a was required for its interaction with TRAF6, we created three truncation mutants: calpain2a (1-339), calpain2a (340-697), and calpain (500-697) (Fig. 3a). HEK293 cells were transfected with calpain2a-Flag wild type (WT), calpain2a-Flag truncation mutants, or TRAF6-HA vector. We found that both full-length calpain2a and three truncation mutants could interact with TRAF6 (Fig. 3c). TRAF6 is composed of a C-terminal TRAF domain, a coiled-coil domain, a Zinc fingers domain, and a Ring finger domain^38^. To examine which domain(s) of TRAF6 was required for its interaction with calpain2a, we made a series of TRAF6 truncates mutants, including TRAF6 (1-139), TRAF6 (140-574), TRAF6 (280-574), and TRAF6 (400-574). Meanwhile, we made the domain mutants: TRAF6ΔRing (in which Ring finger domain is deleted), TRAF6ΔZF (in which Zinc fingers domain is deleted), TRAF6ΔCC (in which coiled-coil domain is deleted), and TRAF6ΔTRAF-C (in which C-terminal TRAF domain is deleted) (Fig. 3b). calpain2a interacted with the full-length TRAF6 as well as truncations of TRAF6 that contained C-terminal TRAF domain, a coiled-coil domain, a Zinc fingers domain (Fig. 3d, f), but not with Ring finger domain (Fig. 3e). Luciferase assays showed that overexpression of calpain2a and these truncation mutants significantly inhibited NF-κB and IL-1β reporter activity in EPC cells (Fig. 3h). Furthermore, the subcellular colocalizations of calpain2a with TRAF6 were investigated. We transfected EPC cells with TRAF6-GFP and an empty vector or calpain2a-mCherry. Confocal microscopy analysis demonstrated that the green signals of TRAF6 were overlapped with the red signals of calpain2a, which indicated that calpain2a co-localizes with TRAF6 in EPC cells (Fig. 3i, j). These data indicate that the structural basis of multiple domains of TRAF6 (C-terminal TRAF domain, coiled-coil domain, Zinc fingers domain) but not Ring finger domain is determinant for its association with calpain2a (Fig. 3g).

### calpain2a inhibits TRAF6 protein expression level by proteolytic activity

**Fig. 4:**
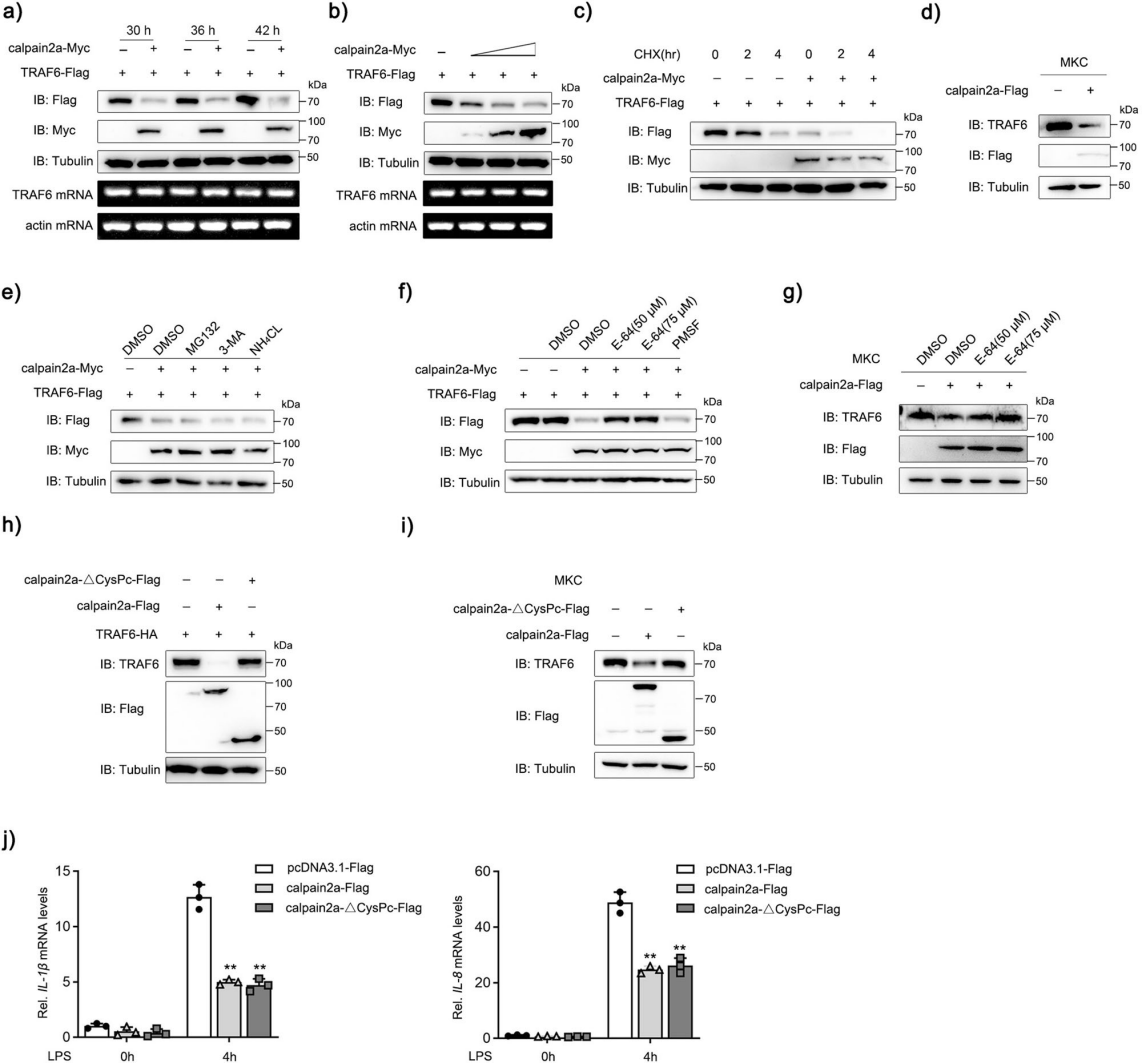
calpain2a inhibits TRAF6 protein expression. **a** The time gradient experiment of empty vectors or calpain2a-Myc plasmids together with TRAF6-Flag was conducted in EPC cells. The cell lysates were subjected to IB with anti-Myc, anti-Flag, and anti–Tubulin Abs. RNA was extracted from cells and reverse transcribed, then TRAF6 and actin were amplified by PCR primers. **b** EPC cells were seeded in 12-well plates overnight and co-transfected with TRAF6-Flag and calpain2a-Myc (0.3, 0.6, or 0.9 μg) for 48 h. The expression of TRAF6-Flag and calpain2a-Myc proteins were detected by Western blotting. RNA was extracted from cells and reverse transcribed, then TRAF6 and actin were amplified by PCR primers. **c** calpain2a-Myc or empty vectors were co-transfected with TRAF6-Flag into EPC cells. At 36 h post-transfection, the transfected cells were treated with cycloheximide (CHX) for 2 or 4 h. **d** MKC cells were transfected with calpain2a-Flag or empty vectors. At 48 h post-transfection, the cell lysates were subjected to IB with anti-TRAF6, anti-Flag, and anti-Tubulin Abs. **e**, **f** EPC cells were transfected with the indicated plasmids in the presence or absence of MG132, 3-MA, NH_4_CL, E-64 (50 or 75 μM), or PMSF for 6 h before immunoblot analysis was performed. **g** MKC cells were transfected with calpain2a-Flag in the presence or absence of E-64 (50 or 75 μM) for 6 h before immunoblot analysis was performed. **h** calpain2a-Flag and calpain2a-ΔCysPc-Flag were co-transfected with TRAF6-HA into EPC cells. At 48 h post-transfection, the cell lysates were subjected to IB with indicated Abs. **i** calpain2a-Flag and calpain2a-ΔCysPc-Flag were co-transfected into MKC cells. At 48 h post-transfection, the cell lysates were subjected to IB with anti-TRAF6, anti-Flag, and anti-Tubulin Abs. **j** *IL-1β*, *IL-8* mRNA in MKC stably transduced with calpain2a-Flag and calpain2a-ΔCysPc-Flag and treated with saline (0) or challenged with LPS for 4 h (*n* = 3 per group). Relative mRNA level was normalized to the expression of the gene encoding *β-actin* in each sample. All experiments were performed in at least three independent experiments. Data were analyzed by two-way ANOVA (**j**). ^**^p  <  0.01.

To explore the regulatory mechanism of calpain2a on TRAF6, we first examined whether calpain2a had an effect on TRAF6 at the protein level. calpain2a-Myc and TRAF6-Flag were co-transfected into EPC cells, the protein level of TRAF6 was partly degraded at 30 h (Fig. 4a). As shown in Fig. 4b, calpain2a promoted the degradation of TRAF6 in a dose-dependent manner. In the presence of calpain2a, it did not affect the mRNA level of TRAF6, indicating that calpain2a only affects the protein level of TRAF6. Meanwhile, the inhibitor cycloheximide (CHX) assay demonstrated that calpain2a could accelerate the half-life of the TRAF6 protein (Fig. 4c). Overexpression of calpain2a in MKC cells resulted in the destabilization of endogenous TRAF6 (Fig. 4d). To investigate whether the degradation of TRAF6 mediated by calpain2a was dependent on the ubiquitin-proteasome, autophagosome, lysosomal pathways, or others, EPC cells were treated with the reagents that inhibited these pathways: MG132, 3-MA, and NH_4_Cl. However, the calpain2a-mediated destabilization of TRAF6 was not reversed by the proteasomal inhibitor MG132, autophagic inhibitor 3-MA, or lysosomal inhibitor NH_4_Cl (Fig. 4e). Next, EPC cells were treated with the reagents: E-64 (a calpain inhibitor which inactivate calpain hydrolase activity) and PMSF (nonspecific serine protease inhibitor). We found calpain inhibitor E-64 prevented degradation at the TRAF6 protein level (Fig. 4f). As shown in Fig. 4g, E-64 also reversed the degradation of endogenous TRAF6 protein levels by calpain2a.

To determine whether the degradation of TRAF6 was dependent on hydrolase activity of calpain2a. Taking advantage of previous structural and biochemical studies of calpain2a in mammals, we mutated the cysteine, histidine, and asparagine residues of calpain2a to alanine (calpain2a C105A, H262A, N286A and 3 A), which lost the catalytic-triad coordination^44–46^. We observed that the degradation of TRAF6 was not affected by this mutation (Supplementary Fig. 1a). So, we mutated the protease domain, which is Calpain family cysteine protease (CysPc) domain of calpain2a (designated as calpain2a-ΔCysPc). Neither overexpressed TRAF6 nor endogenous TRAF6 is affected by calpain2a-ΔCysPc (Fig. 4h, i). However, proinflammatory cytokines *IL-1β* and *IL-8* are simultaneously affected by wild-type or mutated calpain2a upon LPS stimulation (Fig. 4j). Mutant calpain2a lacking hydrolase activity do not affected protein level of TRAF6, but the mutant calpain2a lacking hydrolase activity still affected the expression of downstream pro-inflammatory cytokines. This triggered our investigation into the potential mechanisms by which calpain2a regulates TRAF6. Taken together, these results reveal that calpain2a regulates NF-κB signaling by suppressing the expression of TRAF6 protein levels, through its calpain hydrolase activity.

### calpain2a inhibits TRAF6 ubiquitin-ligase activity

**Fig. 5:**
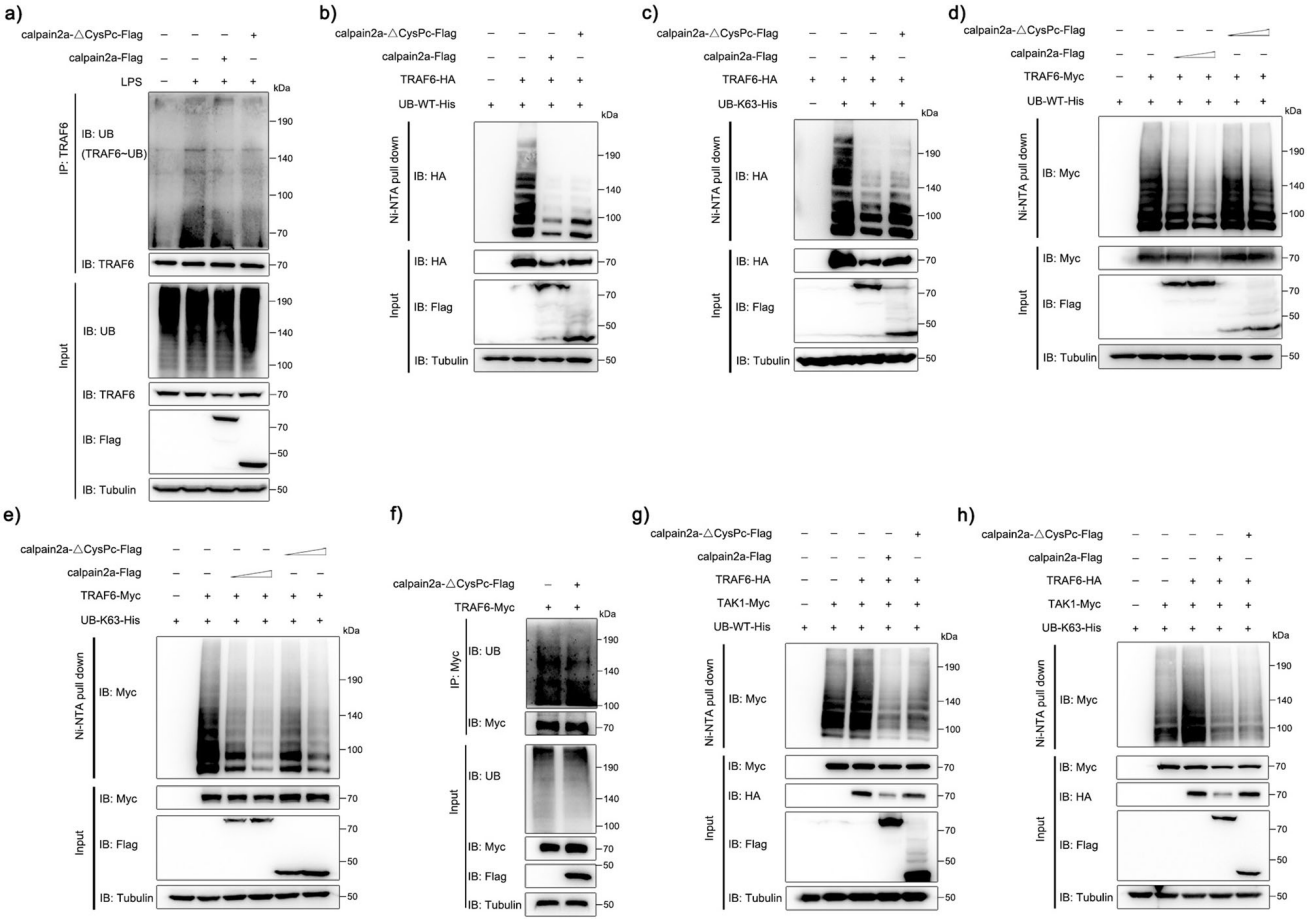
calpain2a inhibits TRAF6 ubiquitin-ligase activity. **a** Ubiquitination of endogenous TRAF6 in MKC cells transduced with calpain2a-Flag and calpain2a-ΔCysPc-Flag and unchallenged (−) or challenged with LPS (+), assessed by immunoblot analysis with anti-ubiquitin after immunoprecipitation with anti-TRAF6 and input immunoblot analysis with indicated Abs. **b**, **c** HEK293 cells were cotransfected with TRAF6-HA and WT-ubiquitin-His or K63O-ubiquitin-His (in which only lysine 63 is kept) together with calpain2a-Flag, calpain2a-ΔCysPc-Flag or empty vector. After 24 h post-transfection, the cells were lysed and purified with Ni-NTA agarose. **d**, **e** HEK293 cells were cotransfected with TRAF6-HA and WT-ubiquitin-His or K63O-ubiquitin-His (in which only lysine 63 is kept) together with calpain2a-Flag (0.5 μg, 1 μg), calpain2a-ΔCysPc-Flag (0.5 μg, 1 μg) or empty vector. After 24 h post-transfection, the cells were lysed and purified with Ni-NTA agarose. **f** Ubiquitination of overexpressed TRAF6 in MKC cells transduced with calpain2a-ΔCysPc-Flag, assessed by immunoblot analysis with anti-ubiquitin after immunoprecipitation with anti-Myc and input immunoblot analysis with indicated Abs. **g**, **h** HEK293 cells were cotransfected with TAK1-Myc, TRAF6-HA and WT-ubiquitin-His or K63O-ubiquitin-His (in which only lysine 63 is kept) together with calpain2a-Flag, calpain2a-ΔCysPc-Flag or empty vector. After 24 h post-transfection, the cells were lysed and purified with Ni-NTA agarose. All the immunoprecipitates and input immunoblot analysis with anti-Myc, anti-Flag, anti-HA, and anti-Tubulin Abs. All experiments were performed in at least three independent experiments.

Next, we explored the molecular mechanisms by which calpain2a negatively regulates TRAF6-activated NF-κB signaling, and whether calpain2a’s interaction with TRAF6 upon loss of enzymatic activity modulates its ubiquitin ligase activity. We treated MKC cells with LPS and then immunoprecipitated TRAF6. Ubiquitination assay with purified recombinant proteins confirmed that calpain2a and calpain2a-ΔCysPc decreased ubiquitination of TRAF6 (Fig. 5a). As shown in Fig. 5b, c, both calpain2a and calpain2a-ΔCysPc significantly reduced the WT and K63 ubiquitinated proteins of TRAF6. Concentration gradients of calpain2a and calpain2a-ΔCysPc reduced WT and K63 ubiquitinated proteins of TRAF6 in a dose-dependent manner. calpain2a-ΔCysPc still prevented K63 ubiquitin chain aggregation of TRAF6 without affecting TRAF6 protein level (Fig. 5d, e). Then we transfected MKC cells to express Myc-tagged TRAF6 in the presence or absence of Flag-tagged calpain2a-ΔCysPc, then performed immunoprecipitation experiments with an anti-Myc antibody and analyzed by immunoblot with anti-UB. Such ubiquitination of TRAF6 was substantially attenuated by calpain2a-ΔCysPc (Fig. 5f). Stimulated by LPS, TRAF6 delivers K63-linked ubiquitin chain to TAK1, which stimulates TAK1 autophosphorylation and activates NF-κB^18^. WT and K63 ubiquitin proteins of TAK1 were significantly enhanced in the presence of TRAF6, whereas TAK1-linked ubiquitination was inhibited in the presence of calpain2a-ΔCysPc (Fig. 5g, h). These data support that mutant calpain2a lacking hydrolase activity similarly inhibits TRAF6 ubiquitin ligase activity without affecting TRAF6 protein levels.

### calpain2a inhibits TRAF6 homo-oligomerization

**Fig. 6:**
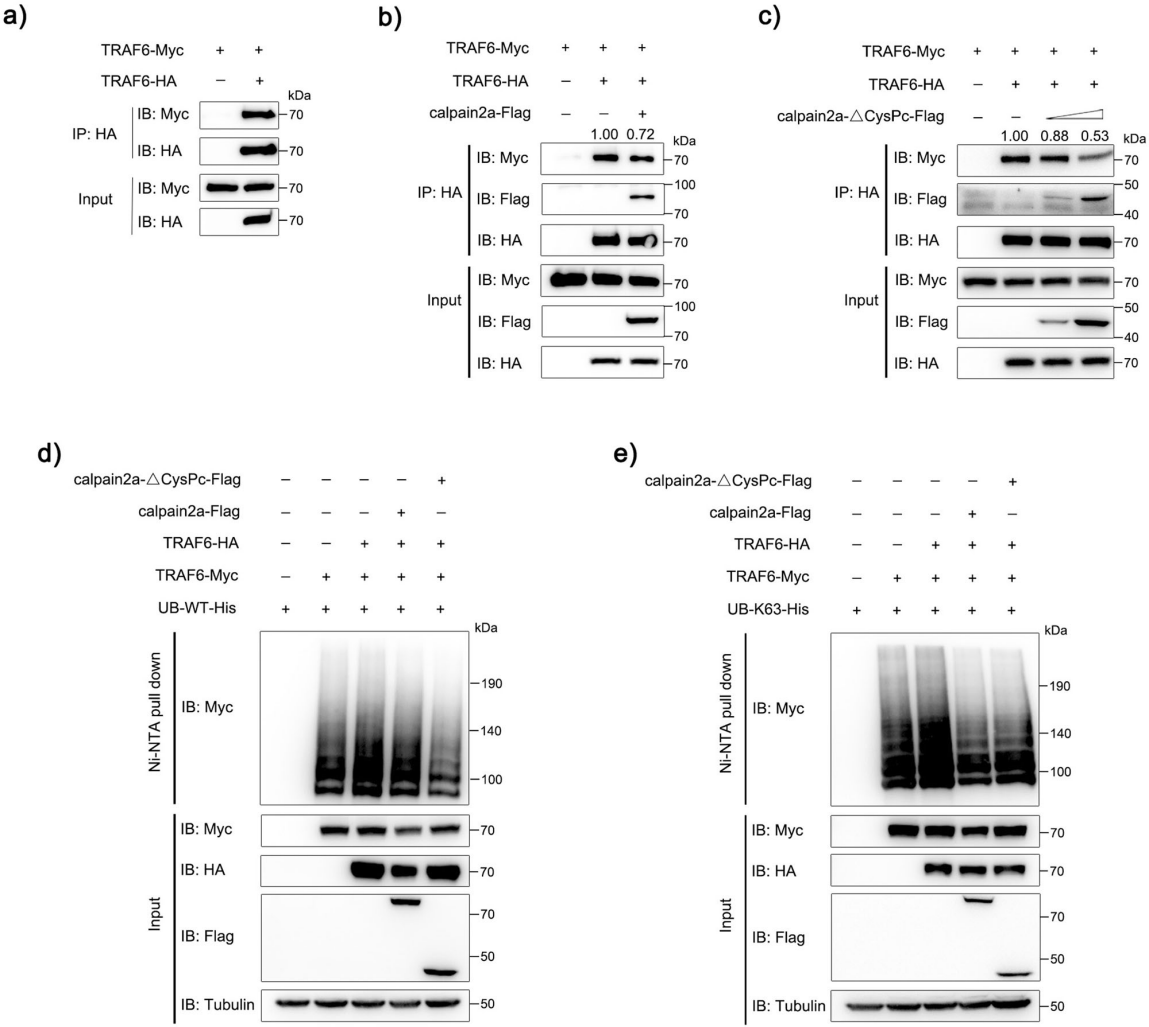
calpain2a attenuates autoubiquitination of TRAF6. **a** HEK293 cells were transfected with TRAF6-Myc, TRAF6-HA, or empty vector. After 24 h post-transfection, the cells were lysed and IP analyses with HA antibody. **b** HEK293 cells were transfected with TRAF6-Myc, TRAF6-HA, empty vector, or calpain2a-Flag. After 24 h post-transfection, the cells were lysed and IP analyses with HA antibody. **c** HEK293 cells were transfected with TRAF6-Myc, TRAF6-HA, empty vector, or different concentrations of calpain2a-ΔCysPc-Flag. After 24 h post-transfection, the cells were lysed and IP analyses with HA antibody. **d**, **e** HEK293 cells were cotransfected with TRAF6-Myc, TRAF6-HA and WT-ubiquitin-His or K63O-ubiquitin-His (in which only lysine 63 is kept) together with calpain2a-Flag, calpain2a-ΔCysPc-Flag or empty vector. After 24 h post-transfection, the cells were lysed and purified with Ni-NTA agarose. The immunoprecipitates and input immunoblot analysis with anti-Myc, anti-Flag, anti-HA, and anti-Tubulin Abs. All experiments were performed in at least three independent experiments.

The Lys63 ubiquitin chain synthesized by TRAF6 plays a key role in signal transduction. It has been reported that the dimerization of TRAF6 is essential for the synthesis of its ubiquitin chain^16^. The dimerization model is closely related to the combination of Ubc13 to produce K63 ubiquitination^16,30^. To verify whether fish TRAF6 could form dimers, we used TRAF6 plasmids with different tags: Myc-tagged TRAF6 and HA-tagged TRAF6 for co-IP experiments. IP assay was performed using anti-HA antibody, HA-TRAF6 interacted with Myc-TRAF6 (Fig. 6a). To explore the inhibitory effect of calpain2a in the TRAF6-dimer interaction, Flag-tagged calpain2a, HA-tagged TRAF6 and Myc-tagged TRAF6 were transiently expressed into HEK293 cells. As expected, the TRAF6-dimer interaction was decreased in the presence of calpain2a (Fig. 6b). At the same time, in order to avoid the presence of the degradation TRAF6 by calpain2a. We transfected Flag-tagged calpain2a-ΔCysPc and found that the TRAF6-dimer interaction was gradually decreased in the presence of calpain2a-ΔCysPc (Fig. 6c). As shown in Fig. 6d, e, we found HA-TRAF6 enhanced WT and K63 ubiquitination of Myc-TRAF6. While HEK293 cells were co-transfected with calpain2a and calpain2a-ΔCysPc, the enhanced ubiquitination would be suppressed. These results suggest that both wild-type calpain2a and mutant calpain2a lacking hydrolase activity inhibits TRAF6 homo-oligomerization and auto-ubiquitination.

### calpain2a inhibits the delivery of TRAF6 ubiquitination to ECSIT and BECN1

**Fig. 7:**
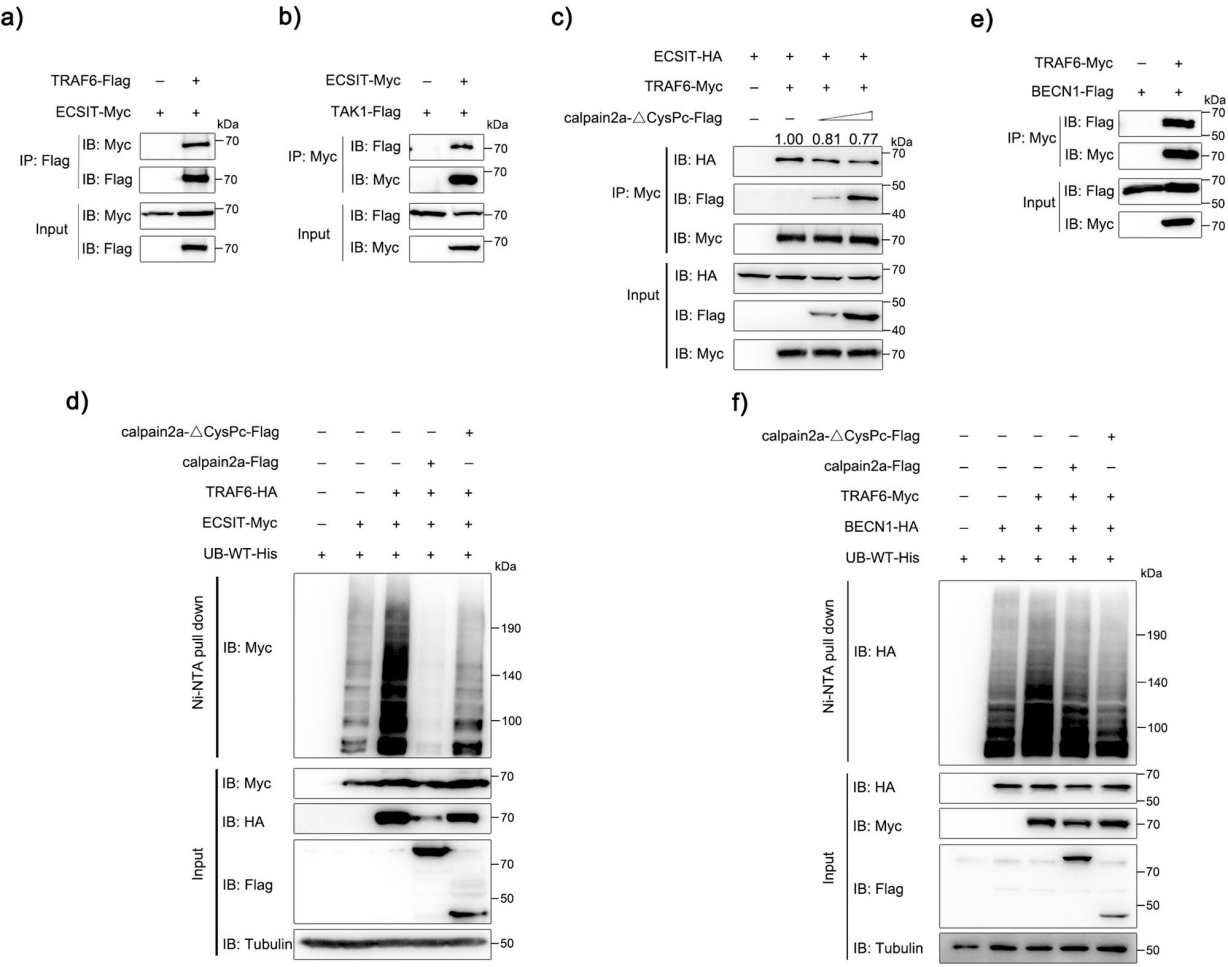
calpain2a inhibits TRAF6-mediated ubiquitination of ECSIT and BECN1. **a** HEK293 cells were transfected with ECSIT-Myc, TRAF6-Flag, or empty vector. After 24 h post-transfection, the cells were lysed and IP analyses with Flag antibody. **b** HEK293 cells were transfected with TAK1-Flag, ECSIT-Myc, or empty vector. After 24 h post-transfection, the cells were lysed and IP analyses with Myc antibody. **c** HEK293 cells were transfected with TRAF6-Myc, ECSIT-HA, empty vector, or different concentrations of calpain2a-ΔCysPc-Flag. After 24 h post-transfection, the cells were lysed and IP analyses with Myc antibody. **d** HEK293 cells were cotransfected with ECSIT-Myc, TRAF6-HA, WT-ubiquitin-His together with calpain2a-Flag, calpain2a-ΔCysPc-Flag or empty vector. After 24 h post-transfection, the cells were lysed and purified with Ni-NTA agarose. **e** HEK293 cells were transfected with BECN1-Flag, TRAF6-Myc, or empty vector. After 24 h post-transfection, the cells were lysed and IP analyses with Myc antibody. **f** HEK293 cells were cotransfected with BECN1-HA, TRAF6-Myc, WT-ubiquitin-His together with calpain2a-Flag, calpain2a-ΔCysPc-Flag or empty vector. After 24 h post-transfection, the cells were lysed and purified with Ni-NTA agarose. The immunoprecipitates and input with anti-Myc, anti-Flag, anti-HA, and anti-Tubulin Abs. All experiments were performed in at least three independent experiments.

As depicted in Fig. 5d, we found that calpain2a interfered with the ubiquitylation process from TRAF6 to TAK1. It has been reported that ECSIT acted as an adaptor protein of TAK1 and TRAF6 to promote NF-κB signaling by LPS stimulation^37^. To verify whether ECSIT had the mentioned functions in teleost fish, we performed multiple sequence alignments of ECSIT proteins in human, mouse, and miiuy croaker (Supplementary Fig. 1b). We transfected EPC cells with NF-κB luciferase reporter, ECSIT significantly enhanced NF-κB reporter activity after LPS stimulation (Supplementary Fig. 1c). Overexpression of ECSIT in MKC cells led to upregulation of the mRNA level of *TNF-α* (Supplementary Fig. 1d). After transfected fish ECSIT and TRAF6 in HEK293 cells, we found that TRAF6 interacted with ECIST (Fig. 7a). Similarly, ECSIT and TAK1 were co-transfected, and the two proteins also maintained interaction (Fig. 7b). Subsequently, to verify whether calpain2a-ΔCysPc inhibits the formation of the TRAF6-ECSIT complex, we transfected the three plasmids into HEK293 cells and found that calpain2a-ΔCysPc prevented the TRAF6-ECSIT complex (Fig. 7c). To verify the transmission process of the ubiquitin chain in NF-κB signaling. As shown in Fig. 7d, TRAF6 could promote the ubiquitination of ECSIT; as expected, calpain2a-ΔCysPc inhibited the process by which TRAF6 transmitted the ubiquitin chain to ECSIT.

BECN1 is an important protein in LPS-induced autophagy. It has been confirmed that K63 ubiquitination could modify and activate BECN1. Deubiquitinating enzyme A20 removes the ubiquitination of BECN1 and inhibit autophagy caused by LPS, and TRAF6 is the E3 ligase responsible for BECN1 ubiquitination in this signal^47^. As shown in Fig. 7e, TRAF6 interacted with BECN1 and promoted the ubiquitination of BECN1. Then we transfected BECN1, TRAF6, calpain2a, and calpain2a-ΔCysPc into HEK293 cells, TRAF6 promoted the ubiquitination of BECN1. calpain2a-ΔCysPc inhibited the process by which TRAF6 transmitted the ubiquitin chain to BECN1 (Fig. 7f). These results suggest that wild-type calpain2a and mutant calpain2a lacking hydrolase activity inhibit the ubiquitination ability of TRAF6 in LPS-induced autophagy and NF-κB signaling.

### Negative regulation of antiviral response by calpain2a

**Fig. 8:**
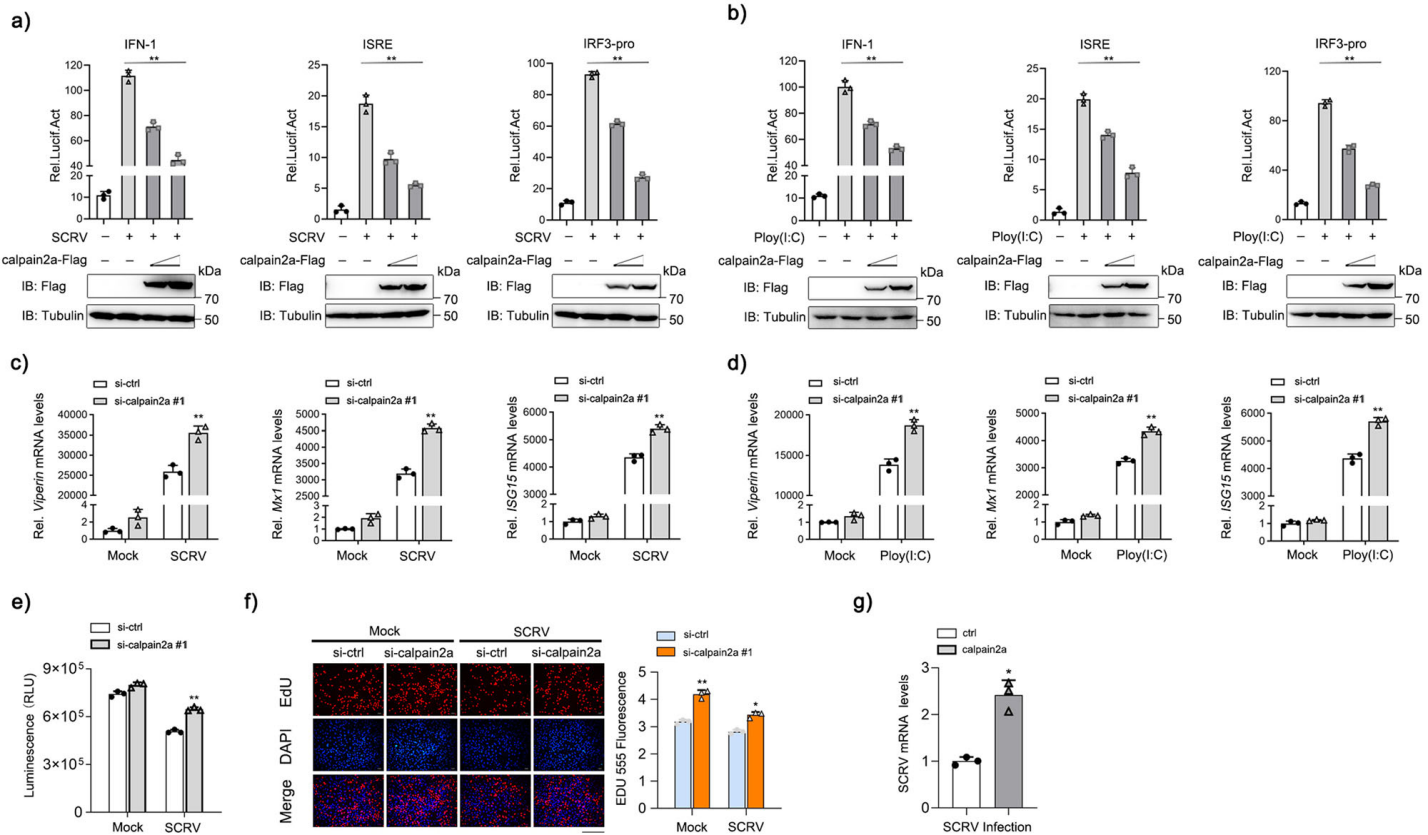
calpain2a inhibits SCRV- or Poly(I:C)-induced IFN activation. **a**, **b** EPC cells were transfected with different concentrations of calpain2a-Flag or empty vector together with the IFN-1, ISRE, and IRF3-pro luciferase reporters. At 24 h post-transfection, cells were mock-infected or infected with SCRV or Poly(I:C) for 12 h (*n* = 3 per group). Western blot analysis was used to measure the expression of transiently transfected calpain2a-Flag. The expression of Tubulin was used as a loading control. **c**, **d** *Viperin*, *Mx1*, *ISG15* mRNA in MKC stably transduced with si-calpain2a #1 or si-ctrl (control vector) and treated with saline (0) or challenged with SCRV or Poly(I:C) for 24 h. Relative mRNA level was normalized to the expression of the gene encoding *β-actin* in each sample (*n* = 3 per group). **e**, **f** MKC cells were transfected with either si-ctrl or si-calpain2a #1. At 24 h post-transfection, the cells were stimulated with SCRV for 24 h, then cell viability assay and cell proliferation assay were measured (*n* = 3 per group). Scale bar, 100 μm. **g** MKC cells were transfected with calpain2a-Flag or pcDNA3.1, then infected with SCRV for 24 h (*n* = 3 per group). The qRT-PCR analysis was conducted for intracellular and supernatant SCRV RNA expression. All experiments were performed in at least three independent experiments. Data were analyzed by one-way ANOVA (**a**, **b**), two-way ANOVA (**c**, **d**, **e**, **f**) or two-tailed Student’s *t*-test (**g**). ^*^p  <  0.05, ^**^p  <  0.01.

In RLR pathway, TRAF6 acts as an E3 ligase recruited by MAVS to activate NF-κB, the production of IFNs and cytokines. We next examined whether calpain2a played a role to prevent TRAF6 in the IFN-mediated antiviral process. In EPC cells, IFN-1, ISRE and IRF3 promoter-driven luciferase reporters were expressed. We found calpain2a substantially reduced these promoter-driven luciferase activities in a dose-dependent manner upon Ploy(I:C) stimulation and Siniperca chuatsi rhabdovirus (SCRV) infection (Fig. 8a, b). Knockdown of calpain2a significantly enhanced Ploy(I:C) and SCRV-triggered gene expression including *Viperin*, *Mx1*, and *ISG15* (Fig. 8c, d). Knockdown of calpain2a resulted in higher expression of cell viability upon SCRV infection in MKC cells (Fig. 8e). As shown in Fig. 8f, cell proliferation assays revealed that knockdown of calpain2a enhanced cell proliferation upon SCRV infection. Overexpression of calpain2a sharply promoted virus propagation, as reflected by viral replication after SCRV infection (Fig. 8g). calpain2a and calpain2a-ΔCysPc substantially reduced IFN-1, ISRE and IRF3 promoter-driven luciferase reporters upon Ploy(I:C) stimulation and SCRV infection (Supplementary Fig. 2a, b). These results suggest that calpain2a may negatively regulate the RLR signaling pathway through inhibition of TRAF6, thereby inhibiting the activation of IFN.

### calpain2a suppresses ubiquitination of IRF3 and IRF7 from TRAF6

**Fig. 9:**
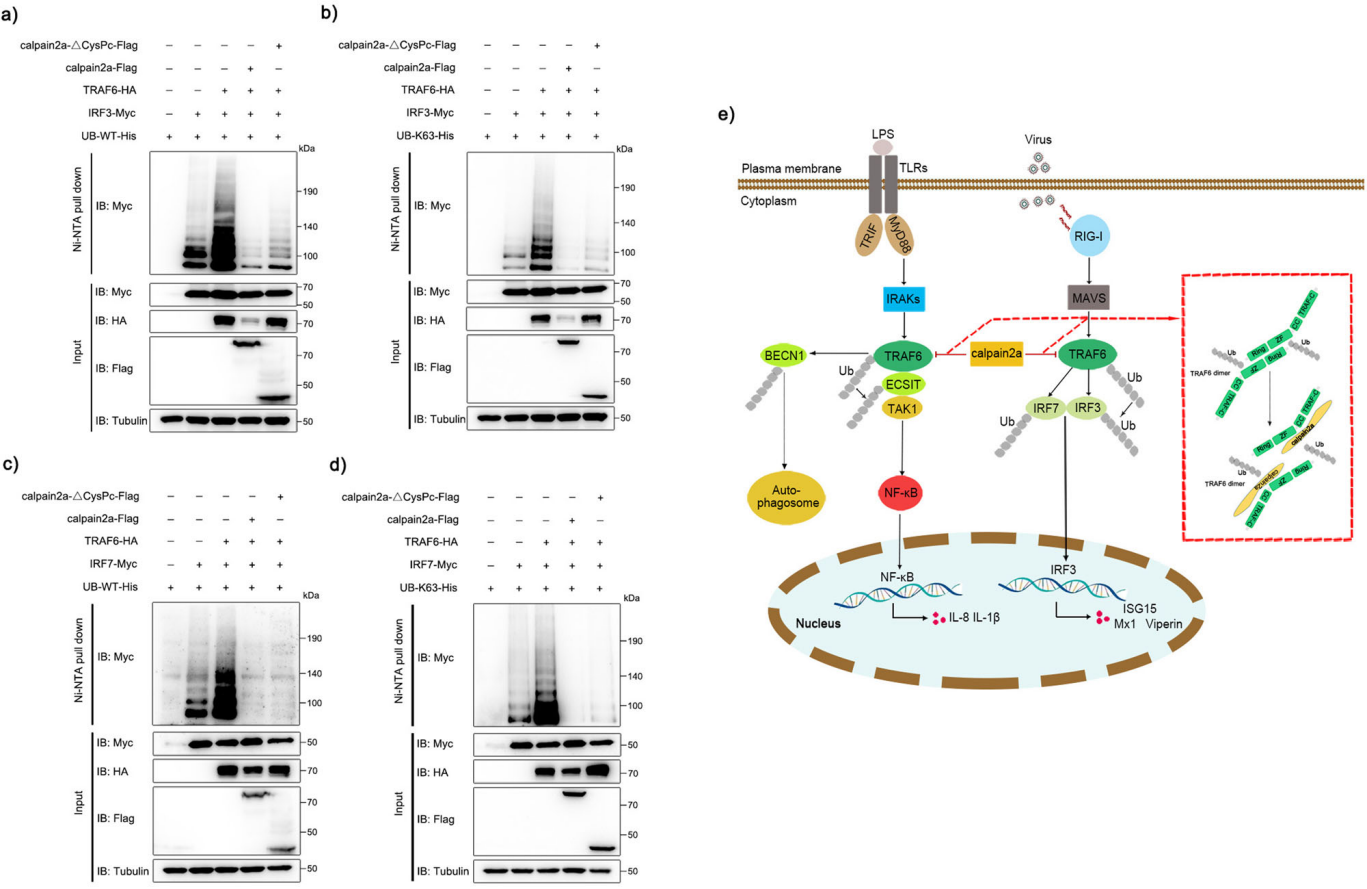
calpain2a inhibits TRAF6-mediated ubiquitination of IRF7 and IRF3. **a**, **b** HEK293 cells were cotransfected with IRF3-Myc, TRAF6-HA and WT-ubiquitin-His or K63O-ubiquitin-His (in which only lysine 63 is kept) together with calpain2a-Flag, calpain2a-ΔCysPc-Flag or empty vector. After 24 h post-transfection, the cells were lysed and purified with Ni-NTA agarose. **c**, **d** HEK293 cells were cotransfected with IRF7-Myc, TRAF6-HA and WT-ubiquitin-His or K63O-ubiquitin-His (in which only lysine 63 is kept) together with calpain2a-Flag, calpain2a-ΔCysPc-Flag or empty vector. After 24 h post-transfection, the cells were lysed and purified with Ni-NTA agarose. All the immunoprecipitates and input with anti-Myc, anti-Flag, anti-HA, and anti-Tubulin Abs. All experiments were performed in at least three independent experiments. **e** Model detailing the roles of calpain2a in TRAF6-mediated signaling pathways. Upon LPS and virus, TRAF6 could be activated, homo-oligomerization and auto-ubiquitination. BECN1, ECSIT, IRF7 and IRF3 are ubiquitinated by TRAF6 and trigger the activation of downstream signals. calpain2a as a negative regulator targets TRAF6 to inhibit TRAF6-mediated signaling pathways.

It is reported that in the antiviral signal triggered by the MAVS-TRAF3/6 complex, the activity of TRAF6 is necessary for the activation of IRF3 and NF-κB^48^. As shown in Fig. 9a, b, TRAF6 promotes WT and K63-linked ubiquitination of IRF3, and this enhancement is inhibited by the expression of calpain2a-ΔCysPc. Meanwhile, when the virus triggers a TLR-dependent signaling signal, TRAF6 is closely related to IRF7 and promotes the ubiquitination of IRF7 along with antiviral factors activated^22,49,50^. TRAF6 promotes WT and K63-linked ubiquitination of IRF7, and this enhancement is inhibited by the expression of calpain2a-ΔCysPc (Fig. 9c, d). These data suggest that wild-type calpain2a and mutant calpain2a lacking hydrolase activity inhibit TRAF6-mediated ubiquitination of IRF3 and IRF7.

## Discussion

TRAF6 is one of the seven members of the Tumor necrosis factor (TNF) receptor-associated factors (TRAFs) family, which has been identified as signal adaptors and exerts signal transduction^51,52^. TRAF family contains the Ring finger domain, which owns the E3 ligase activity. E3 ligases with the HECT domain promote substrate polyubiquitination, E3s with the Ring domain require E2~ub to transmit polyubiquitination^53,54^. The dimer Ub-binding enzyme Ubc13/Uev1A has been confirmed to be involved in the aggregation of TRAF6 polyubiquitination^16^. While K124 of mouse TRAF6 is knocked out, TRAF6 will lose the activity of K63 auto-ubiquitination, and the mutation at this site eliminates the ubiquitination of TRAF6-mediated NEMO and the activation of TAK1, IKK, and NF-κB^55^. It is precise because PRDX1 binds to the TRAF6 Ring Finger domain contained K124 and eliminates the ubiquitination of TRAF6^38^. It has been reported the ubiquitination of TRAF6 is closely related to the dimerization of its N-terminal domain (Ring finger and ZF fingers domain). Multiple N-terminal interaction sites with Ubc13 were mutated, Ubc13 could not transmit poly~ub to TRAF6^16^.

The interaction between calpain2a and TRAF6 mutants showed that most of the domains of TRAF6 interacted with calpain2a, except for the Ring finger domain. Due to the interaction between calpain2a and ZF fingers domain of TRAF6, we speculated that calpain2a might inhibit auto-ubiquitination by blocking the dimerization of TRAF6. Subsequently, we verified the interaction of different tags of TRAF6. As the expression of mutant calpain2a lacking hydrolase activity increased, the interaction of different tags of TRAF6 weakened. HA-tagged TRAF6 promotes the ubiquitination of Myc-tagged TRAF6, and mutant calpain2a inhibits the ubiquitination enhanced by HA-tagged TRAF6. As an intermediate in the process of TRAF6-TAK1 signal transmission, ECSIT binds to the C-terminal domain of TRAF6 (CC domain and TRAF-C domain) and is confirmed to be involved in NF-κB signaling^37^. The expression of proinflammatory cytokines decreased in ECSIT^KD^ THP-1 cells. However, after overexpression of ECSIT in ECSIT^KD^ THP-1 cells, genes such as IRF7, IL-1β, and CD44 increased significantly^37^. PRDX1 binds to Ring finger domain of TRAF6 and inhibits the ubiquitination of ECSIT and BECN1 in NF-κB and autophagy signals by inhibiting the formation of TRAF6 polyubiquitination^38^. In teleost fish, we confirmed ECSIT activated NF-κB signaling pathway, interacted with TRAF6 and TAK1 respectively, and transmitted the polyubiquitin chain produced by TRAF6. calpain2a bound to ZF domain, CC domain, and TRAF-C domain of TRAF6, which inhibited the interaction of TRAF6-ECSIT. We confirmed that mutant calpain2a lacking hydrolase activity prevented the polyubiquitin chain from TRAF6 to ECSIT and BECN1.

We notice that calpain2a has hydrolase activity as one of the calcium-dependent proteases family. m-calpain (calpain-2) plays a positive regulatory role in NF-κB signaling pathway of HepG2 liver cells. Independent of the 26 s proteasome approach, calpain2a hydrolyzes IkBα to release the transcription factor NF-κB. The specific inhibitor of calpain protein prevents the degradation of IkBα^56^. However, for calpain, there are few studies in fish innate immune response. In MKC cells, overexpression of calpain2a inhibited NF-κB downstream proinflammatory cytokines induced by LPS. This phenomenon was contrary to the results in HepG2 liver cells. It might be that the species-specificity of calpain protein family caused different effects. Similarly, we used the specific inhibitor of the calpain protein E-64 to prevent calpain2a from hydrolyzing TRAF6. Mutant calpain2a lacking hydrolase activity inhibits TRAF6 ubiquitin ligase activity without affecting its protein levels. We did not find enzyme-active sites for calpain2a. According to previous studies in mammals, the three sites: cysteine (C105A), histidine (H262A), and asparagine residues (N286A). However, our experiments confirm that the three active sites of miiuy croaker calpain2a do not affect the expression of TRAF6 protein levels, but mutant calpain2a lacking hydrolase domain does. Therefore, we chose to mutate the entire enzyme-activating structural domain. Although protein calpain2a is relatively conserved in mammals and lower vertebrate, the specific enzymatic activity sites deserve further investigation.

In summary, we identified calpain2a as a negative regulator of LPS-induced NF-κB signaling and virus-induced IFN activation (Fig. 9e). In mass spectrometry, calpain2a may interact with TRAF6 and we demonstrated this phenomenon by endogenous coimmunoprecipitation experiment. We found that calpain2a is involved in regulating the LPS-induced NF-κB signaling pathway. In addition, TRAF6 also plays an important role in antiviral signaling pathways. We used SCRV and Ploy(I:C) to stimulate IFN signaling pathway and found that calpain2a may also inhibit IFN signaling by regulating protein level and autoubiquitination of TRAF6. Since TLR3 can recognize the double-stranded RNA (dsRNA) structure produced by viral infections. We are more interested in elucidating calpain2a regulation of TRAF6-mediated IFN signaling pathway, but do not exclude a potential relationship between calpain2a and TLR3 signaling pathway. calpain2a promoted degradation of TRAF6 in a hydrolytic manner and interacted with TRAF6 to inhibit the formation of dimers and auto-ubiquitination. This inhibition prevented the interaction of TRAF6 and ECSIT and the ubiquitination transfer process from TRAF6 to ECSIT, BECN1, IRF3 and IRF7. Our current results will contribute to the understanding of the negative regulation mechanisms by targeting TRAF6 in innate immune responses. In addition, this research indicates the key role of TRAF6 homo-oligomerization and auto-ubiquitination in the process of fish innate immune response. Concomitantly, calpain2a is proposed as a regulatory mechanism protein to inhibit the immune response of TRAF6 and prevent the normal and orderly signal pathways. The insights are helpful to understand vertebrate immunology and the evolution of the vertebrate immune system.

## Methods

### Cell culture

Human embryonic kidney (HEK) 293 cells lines and Epithelioma papulosum cyprini (EPC) cells lines, purchased from American Type Culture Collection and kept in our lab. Miiuy croaker (*M. miiuy*) kidney cell lines (MKC) and Miiuy croaker intestine cell lines (MIC) were cultured from kidney and intestine tissues of miiuy croaker^57,58^. Miiuy croaker kidney cell lines and Miiuy croaker intestine cell lines were cultured at 26 °C in a humidified incubator containing 0.5% CO_2_ in L-15 medium (HyClone, USA) supplemented with 15% FBS (Gibco, USA), 100 U/ml penicillin (Gibco, USA), 100 μg/ml streptomycin (Gibco, USA), and 20 U/ml heparin (Solarbio, China). Epithelioma papulosum cyprini cells were grown at 26 °C in a humidified incubator containing 5% CO_2_ in medium 199 (Hyclone) supplemented with 10% FBS, 2 mM L-glutamine, 100 U/ml penicillin, and 100 mg/ml streptomycin. Human embryonic kidney 293 cells were grown at 37 °C in a humidified incubator containing 5% CO_2_ in Dulbecco’s modified Eagle’s medium (DMEM) (Invitrogen) supplemented with 10% FBS, 2 mM L-glutamine, 100 U/ml penicillin, and 100 mg/ml streptomycin.

### Plasmid construction and reagents

Using the cDNA of MKC cells as a template, the open reading frame (ORF) of miiuy croaker calpain2a (GenBank accession number ON164842) was generated by PCR and then cloned into pcDNA3.1(+) (Invitrogen) with Myc, Flag, and HA tag. The ORF of miiuy croaker TRAF6 (GenBank accession number MZ360962) was generated by PCR and then cloned into pcDNA3.1(+) (Invitrogen) with Myc, Flag, and HA tag. The ORFs of miiuy croaker TAK1 (GenBank accession number ON164843), IRF7 (GenBank accession number ON164846), ECSIT (GenBank accession number ON164844), and IRF3 (GenBank accession number ON164845) were cloned into pcDNA3.1(+) (Invitrogen) with Myc tag. The ORF of miiuy croaker BECN1 was cloned into pcDNA3.1(+) (Invitrogen) with Flag and HA tag. For subcellular localization, the ORF of miiuy croaker TRAF6 was inserted into pEGFP-N1 (Invitrogen), the ORF of miiuy croaker calpain2a was cloned into pCS2-mCherry vector (Clontech). All primers used for PCR are shown in Supplementary Table 1.

The proteasome inhibitor used at a final concentration: MG132 (20 μM) (Sigma), 3-MA (2 mM) (Sigma), NH_4_Cl (20 mM) (Sigma), E-64 (50 μM) (Solarbio), PMSF (100 μM) (Beyotime), and the cycloheximide (CHX) (Beyotime) used at a final concentration of 100 μg/ml.

### Transient transfections and treatment

The EPC cells were seeded into 12-well plates or 48-well plates and incubated overnight. The HEK293 cells were seeded into 6 cm^2^ dishes overnight. Lipofectamine™ RNAiMAX (Invitrogen) was used for transient transfection of si-ctrl, si-calpain2a into MKC cells. DNA plasmid transfection was performed on the cells using Lipofectamine™3000 (Invitrogen) according to the manufacturer’s instructions. Lower vertebrates, such as teleost fish, are resistant to the toxic effects of LPS, so high concentrations of LPS (10 μg/ml) have been used to activate immune cells^59^. For stimulation experiments, MKC and EPC cells were challenged with 10 μg/ml LPS at different times for RNA extraction. For infection experiments, MKC and EPC cells were challenged with 10 μg/mL Poly (I:C) and *Siniperca chuatsi* rhabdovirus (SCRV) at a MOI of 5 and harvested at different times for RNA extraction.

### Luciferase reporter assay

EPC cells were seeded in 48-well plates and transfected with different vectors, including NF-κB-Luc, IL-1β-Luc, IL-8-Luc, IFN-1-Luc, ISRE-Luc, and IRF3-pro-Luc. Construction of reporter genes was performed as previously described^60,61^. The *Renilla* luciferase reporter plasmid (pRL-TK, Promega) was regarded as the internal control. After 24 h post-transfection, cells were treated with or without the addition of LPS for 6 h and lysed. Poly (I:C) and SCRV were performed 24 h before cell harvest. Cells were washed with PBS and lysed with Cell Lysate Buffer (CLB). Luciferase activity was measured with the Dual-Luciferase Reporter Assay System according to the manufacturer’s instructions (Promega)^62^.

### RNAi experiments

For transient silencing, MKC cells were seeded in 12-well plates overnight and transfected with 75 nM si-ctrl and siRNA. Miiuy croaker calpain2a-specific siRNA (si-calpain2a) sequences were GAGACCCACGGAACUUGUUUCUAAU (si-calpain2a #1) and CACGGAACUUGUUUCUAAUCCUGAA (si-calpain2a #2). Nontargeting siRNA “UUCUCCGAACGUGUCACGUTT” was used as a negative control (si-ctrl).

### RNA isolation and qRT-PCR analysis

Total RNA was isolated with TRIzol reagent (Takara) according to the manufacturer’s instructions. First-strand cDNA was synthesized using FastQuant RT Kit (Tiangen) which included DNase treatment of RNA to eliminate genomic contamination. qRT-PCR was performed with SYBR Premix Ex Taq Kit (TaKaRa) on the QuantStudio 3 real-time PCR system (Thermo Fisher Scientific). PCR conditions were as follows: 10 s at 95 °C, followed by 40 cycles of 5 s at 95 °C, and then 31 s at 60 °C^63^. All primers used for qRT-PCR are shown in Supplementary Table 2. and the *β-actin* gene was used as an internal control. The relative fold changes were calculated by comparison to the corresponding controls using the 2^-ΔΔCt^ method. Three independent experiments were conducted for statistical analysis.

### co-IP and Western blot analysis

For co-IP and western blot analysis, 10 μg total plasmids were co-transfected into HEK293 cells, which were collected 20 h after transfection and lysed in IP buffer (1% NP-40, 50 mM Tris-HCl, pH7.4, 50 mM EDTA, 150 mM NaCl) containing protease inhibitor cocktail (Sigma). After centrifugation at 12000 rpm for 15 min at 4 °C, supernatants were collected and incubated with Protein A/G PLUS-Agarose (Santa Cruz) together with monoclonal anti-Myc, anti-Flag, or anti-HA (Abcam). After 8 h at 4 °C with soft agitation, beads were washed four times with the IP buffer above-mentioned and resuspended in 75 µl 2× SDS loading buffer. The immunoprecipitates and whole-cell lysates (WCLs) were analyzed by IB with the indicated antibodies (Abs). For western blot analysis, WCL were separated by 10% SDS-PAGE and transferred to polyvinylidene difluoride (PVDF) membrane (Millipore) and then blotted^64,65^.

The Abs used were as follows: anti-Myc (1:1000), anti-Flag (1:1000), anti-HA (1:1000), anti-Tubulin (1:1000), and anti-mCherry (1:1000) were from Abcam. Endogenous antibodies, anti-MyD88 (1:500), anti-IRAK4 (1:500), anti-TRAF6 (1:500), anti-TAK1 (1:500), anti-calpain2a (1:500) and anti-ubiquitin (1:500) were purchased from Boster Biological Technology.

### Ubiquitination assay

For analysis of the ubiquitination in HEK293 cells, transfected HEK293 cells were washed twice with PBS. Then add 700 µl PBS, HEK293 cells were scraped with cell scraper and collected into a centrifuge tube, centrifuged at 1000 *g* for 5 min. After aspirating 100 µl of PBS containing cells, lysed with NP-40 lysis buffer for Input analysis. Remaining cells were lysed using Lysate containing urea and sonicated. Denatured cell extracts purified with Ni-NTA agarose (Denaturation type). For analysis of endogenous ubiquitination of TRAF6 in MKC. MKC cells were extracted with 60 μl PBS containing 1% SDS, 95 °C for 10 min. After being added to 600 µl NP-40 IP buffer for lysed, supernatants were collected and incubated with Protein A/G PLUS-Agarose (Santa Cruz), then followed by immunoprecipitating with anti-TRAF6 Ab. The immunoprecipitants were analyzed by immunoblotting with anti-ubiquitin Ab.

### Affinity purification and mass spectrometry (MS)

EPC cells were transfected with TRAF6-Flag and pcDNA3.1-Flag as an additional control. We purified TRAF6-Flag from EPC cells lysed by NP-40 Lysis Buffer using a single chain anti-Flag antibody coupled to agarose beads. We use control agarose beads to pull down the same lysate in parallel to control non-specific binding. The purified protein complex is separated on SDS-PAGE and silver stained. In pulldown experiment, 160 proteins were significantly enriched in TRAF6-Flag pulldowns compared with the normal bead control by using affinity purification mass spectrometry. The protein in the TRAF6 database of potentially related proteins has a maximum LFQ intensity of 693.8 × 10^6^. The LFQs in the control samples is 0 and we intercepted part of the data showed in Fig. 1a.

### Fluorescent microscopy

EPC cells were grown on glass coverslips in 24-well plates overnight, fixed with 4% PFA (Beyotime). After being washed three times with PBS and treated with 0.1% Triton X-100 (Sigma) to permeabilize for 15 min. After three washes in PBS, EPC cells were stained with 1 μg/ml DAPI (Beyotime) for 15 min in the dark at room temperature. Finally, the coverslips were washed and observed with a confocal microscope under a ×63 oil immersion objective (SP8; Leica).

### Cell viability and proliferation

MKC cells were grown on glass coverslips in 24-well plates and transfected with siRNA. Cell viability was measured at 48 h after transfection in MKCs with CellTiter-Glo Luminescent Cell Viability assays (Promega) according to the instructions^61^. Cell proliferation assays were performed with BeyoClickEdU cell Proliferation Kit with AlexaFluor 488 (Beyotime) following the instructions^61^.

### Statistics and reproducibility

All data are presented as mean ± SE (standard error) of three experiments. Statistical significance was determined with Student’s *t*-test, one-way ANOVA or two-way ANOVA (two-sided), with the *p* values <0.05 (*) and <0.01 (**) considered statistically significant.

### Reporting summary

Further information on research design is available in the Nature Portfolio Reporting Summary linked to this article.

## Supplementary information

Supplementary Information

Description of Additional Supplementary Files

Supplementary Data 1

Reporting Summary

## Data Availability

The data that support the findings of this study are available in the methods and/or supplementary material of this article. Uncropped Western blot images are provided in Supplementary Figure 3-6. The mass spectrometry data were deposited at Figshare with a 10.6084/m9.figshare.22194220.v1. All statistical source data that underlie the graphs in figures are provided in Supplementary Data 1.
