# Supplementary material for: The protease calpain2a limits innate immunity by targeting TRAF6 in teleost fish
Source: Commun Biol. 2023 Mar 31;6:355. doi: 10.1038/s42003-023-04711-7 (PMC10066338; doi:10.1038/s42003-023-04711-7)
Supplement: Supplementary file 1 — Supplementary Information [file 42003_2023_4711_MOESM1_ESM.pdf]

Supplementary Information

The protease calpain2a limits Innate Immunity by Targeting TRAF6 in Teleost Fish

Yang Chen[1], Pengfei Wang[1], Qi Li[1], Xiaolong Yan[1], Tianjun Xu[1,2,*]

The supplementary information includes:

Supplementary Figure 1-6 and Supplementary Table 1, 2

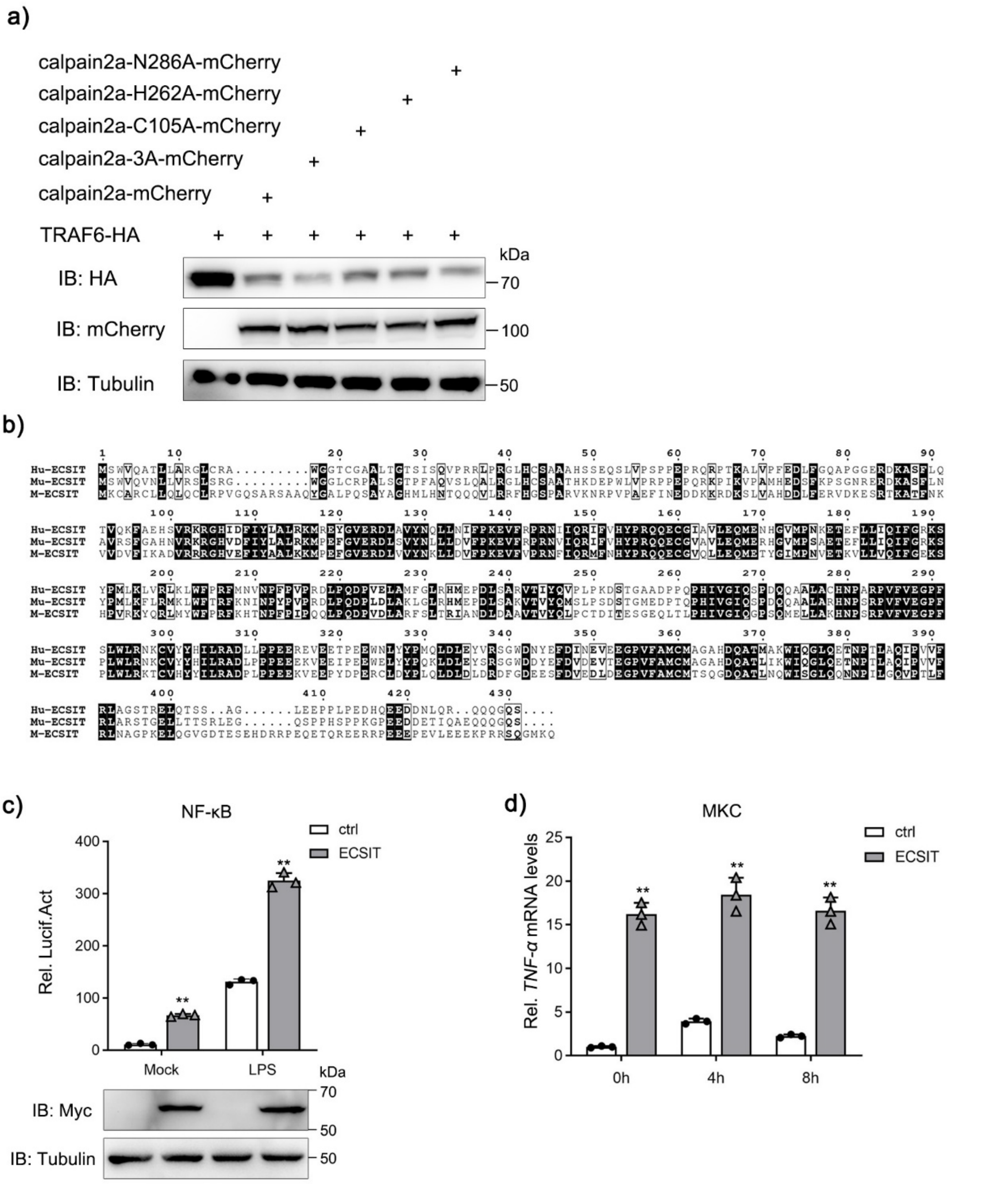

Supplementary Figure 1. ECSIT activates LPS-induced NF-κB Signaling Pathway. a calpain2a-mCherry and the point mutation inactivating mutant (calpain2a-C105A, H262A, N286A, and 3A) were co-transfected with TRAF6-HA into EPC cells. At 48 h post-transfection, the cell lysates were subjected to IB with indicated Abs. **b** The same amino acids among human ECSIT (Hu-ECSIT), mouse ECSIT (Mu-ECSIT), and miiuy croaker ECSIT (M-ECSIT) are highlighted with black background. **c** EPC cells were transfected with ECSIT-Myc or empty vector together with the NF-κB luciferase reporters. At 24 h post-transfection, cells were untreated (Mock) or treated with LPS for 6 h. The

luciferase activity value was achieved against the Renilla luciferase activity ($n = 3$ per group). Western blot analysis was used to measure the expression of transiently transfected ECSIT-Myc. The expression of Tubulin was used as a loading control. **d** *TNF-α* mRNA in MKC stably transduced with ECSIT or empty vector and treated with saline (0) or challenged with LPS for various times (4 h, 8 h) ($n = 3$ per group). Relative mRNA level was normalized to the expression of the gene encoding β-actin in each sample. All experiments were performed in at least three independent experiments. Data were analyzed by two-way ANOVA (c, d). ** $p < 0.01$.

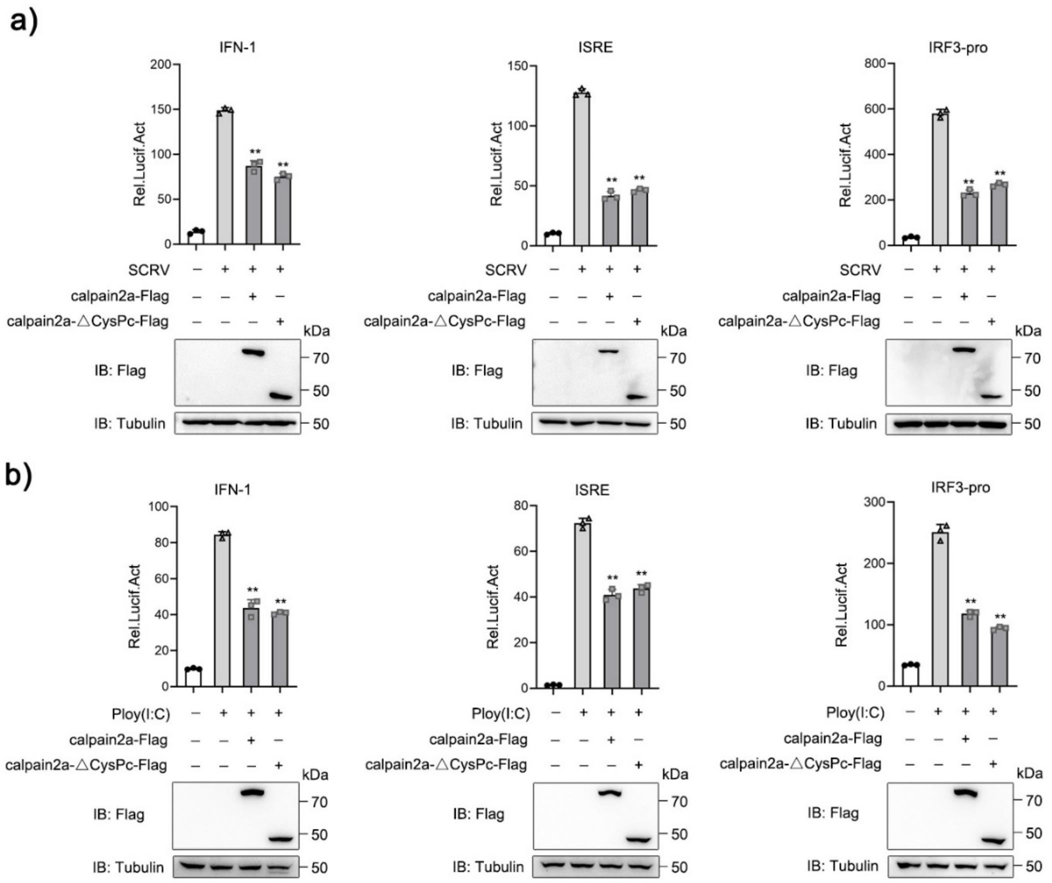

Supplementary Figure 2. Inactive calpain2a mutant inhibits SCRV- or Poly(I:C)-induced IFN activation. a, b EPC cells were transfected with calpain2a-Flag or calpain2a-△CysPc-Flag together with the IFN-1, ISRE, and IRF3-pro luciferase reporters. At 24 h post-transfection, cells were mock-infected or infected with SCRV **a** or Poly(I:C) **b** for 12 h (n = 3 per group). Western blot analysis was used to measure the expression of transiently transfected calpain2a-Flag and calpain2a-△CysPc-Flag. The expression of Tubulin was used as a loading control. All experiments were performed in at least three independent experiments. Data were analyzed by one-way ANOVA (a, b). * $p < 0.05$, ** $p < 0.01$.

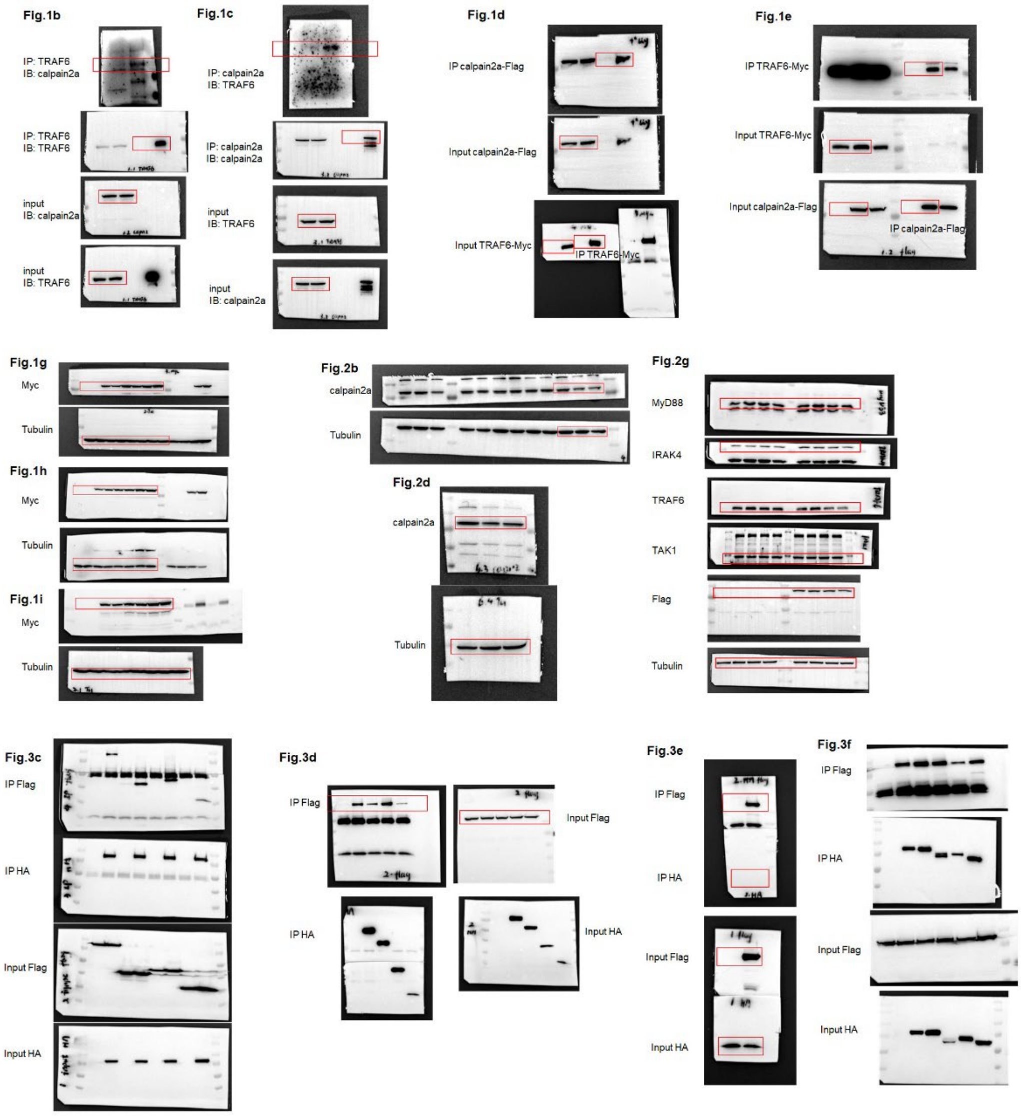

Supplementary Figure 3. Uncropped scans of western blots for Fig1, Fig2, Fig3.

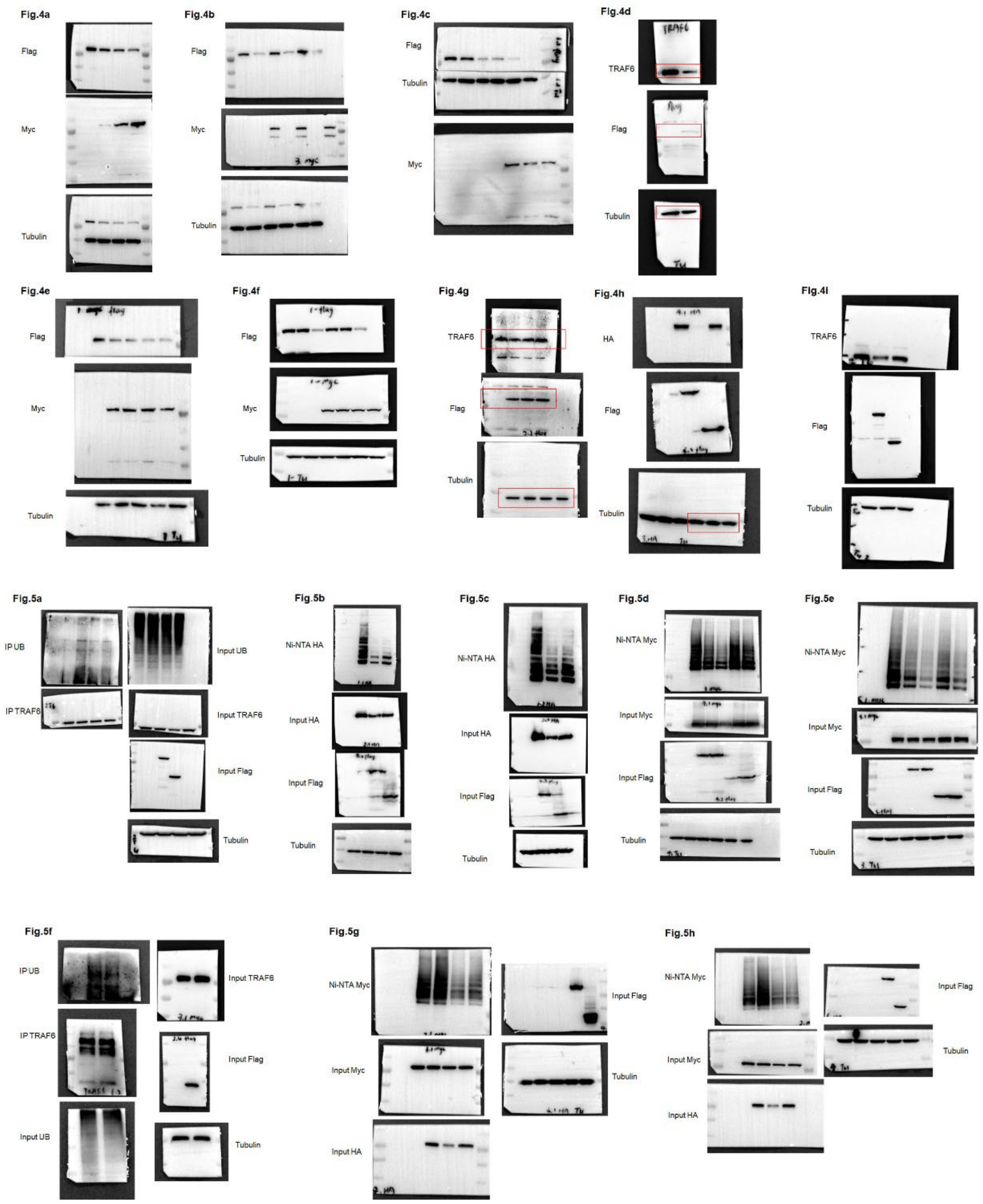

Supplementary Figure 4. Uncropped scans of western blots for Fig4, Fig5.

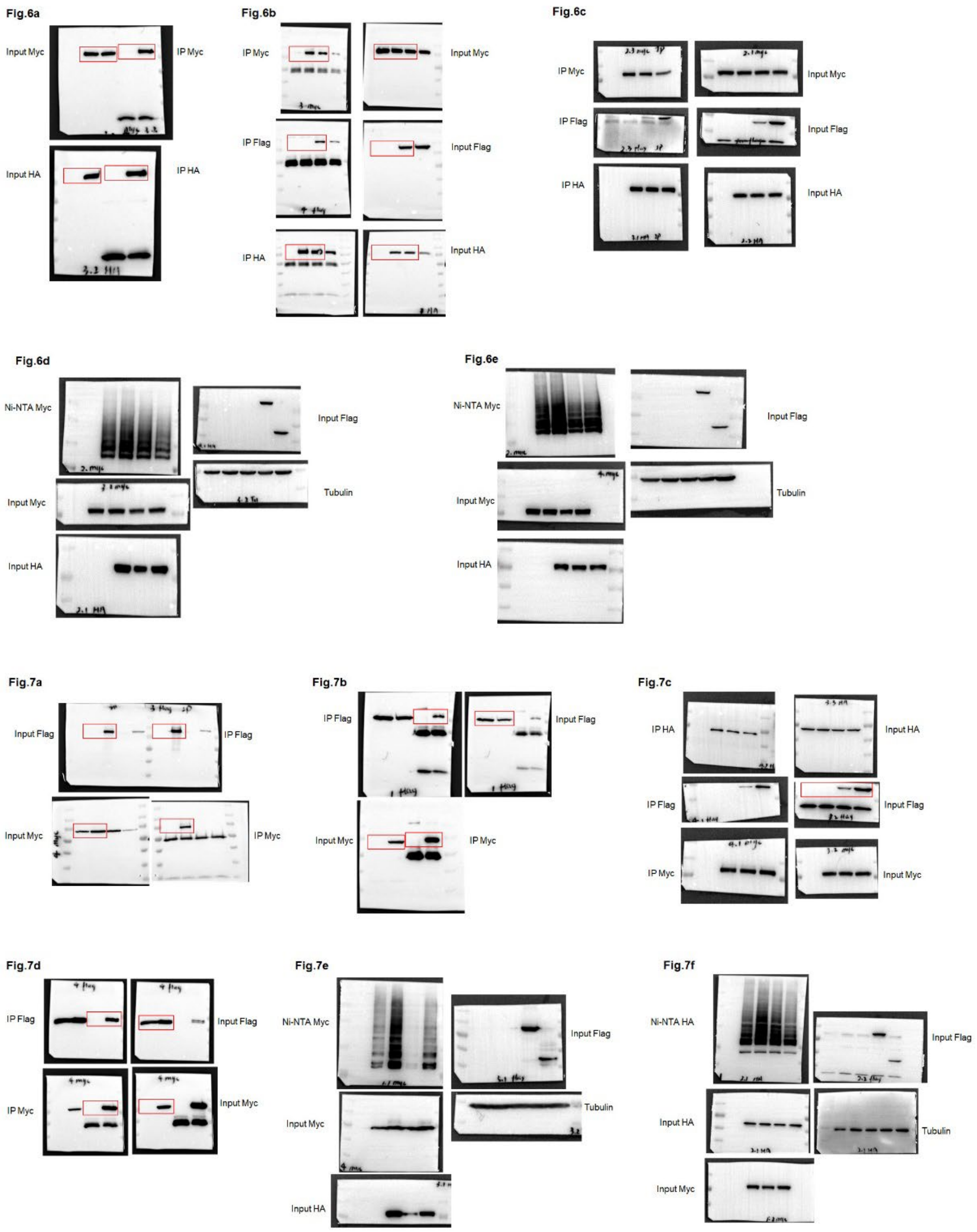

Supplementary Figure 5. Uncropped scans of western blots for Fig6, Fig7.

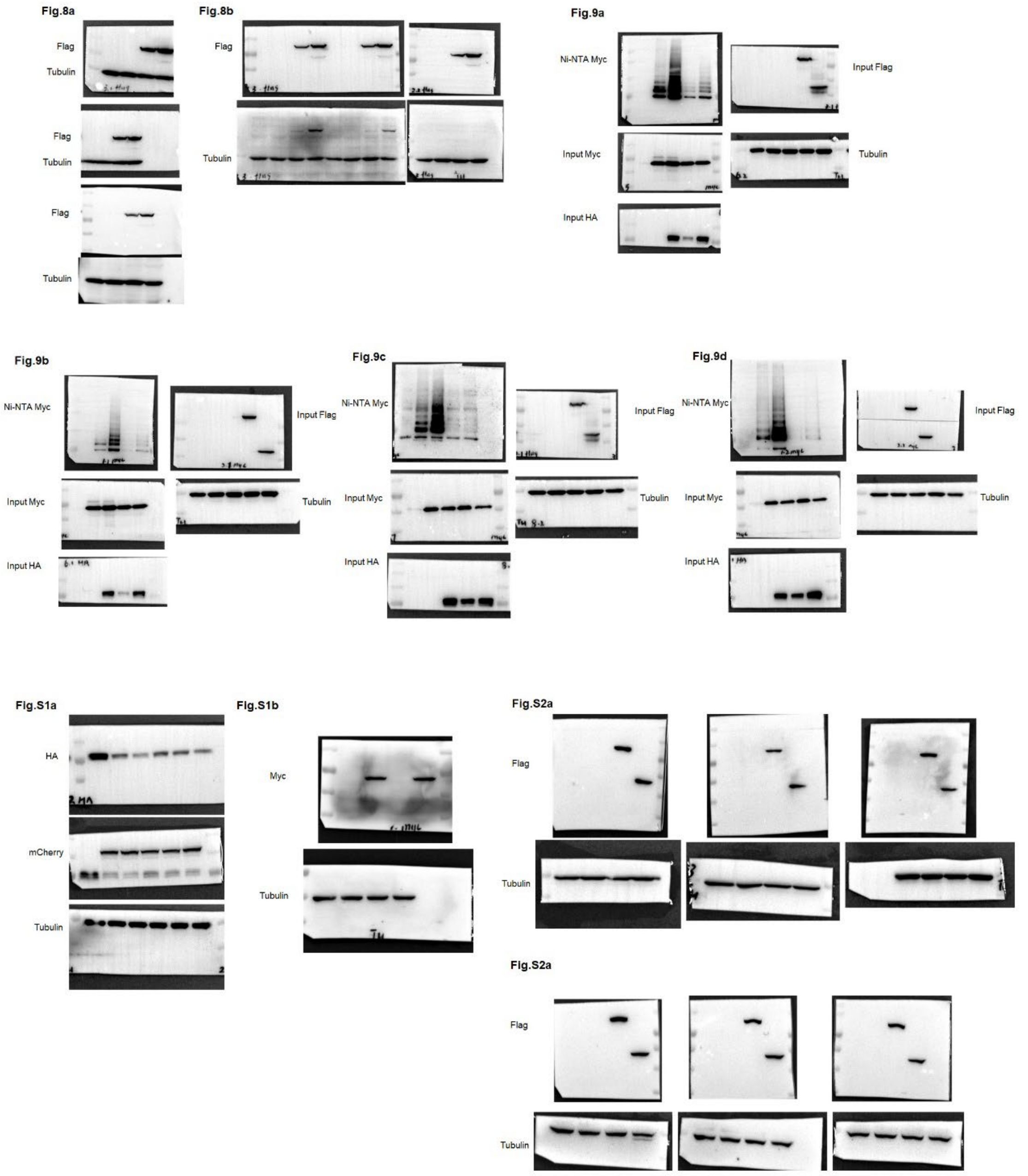

Supplementary Figure 6. Uncropped scans of western blots for Fig8, Fig9, FigS1, FigS2.

Supplementary Table 1: List of primers for vector construction and in this study.

Primer name	Sequences (5'-3')
TRAF6-F	CGCGGATCCATGGCTTGCATTGACAGC
TRAF6-R	CCGGAATTCTTTCCCCATTCTGGTGTC
calpain2a-F	CCCAAGCTTATGTCTGGCGTGGCTTCC
calpain2a-R	CCGGAATTCCTGTCAGTCAGGGCGTGT
ECSIT-F	CCCAAGCTTTCTACCGGCTCTACCATGA
ECSIT-R	CGCGGATCCCTGCTCACTGTTTCATCCC
BECN1-F	CCCAAGCTTCCTTCGCAAGCGACACTAGC
BECN1-R	CCGGAATTCTGAGGCAAAGTCCAGCAGGT
TAK1-F	CTTGGTACCGAGCTCGGATCCATGTCTCTAACGTTACCGTCCGC
TAK1-R	TGATGGATATCTGCAGAATTCGACATGCAGGACACAGTAGAATGC
IRF7-F	CGCGGATCCATGCAAAGCCTTCCAAAGCC
IRF7-R	GCTCTAGATTCTTAATAAAGCTCAGCAGCCA
IRF3-F	CCCAAGCTTATGTCTCATTCTAAACCTCTGC
IRF3-R	CCGGAATTCTGGTTTTCTTAAGGCACAG
calpain2a (1-339)-F	GTGCACTCTGATGATCTGAGCCATTGCTCCGTG
calpain2a (1-339)-R	CAGATCATCAGAGTGCACACCTCAATACGAGAG
calpain2a (340-697)-F	ATTCGGCCTGACTCCTGACGCCATCGAAGATGA
calpain2a (340-697)-R	TCAGGAGTCAGGCCGAATCCAGCTGCCAGGGCC
calpain2a (500-697)-F	TGGATTCGGCTCCGAGAAGCAGTCCGAGACCCA
calpain2a (500-697)-R	TTCTCGGAGCCGAATCCAGCTGCCAGGGCCCGC
TRAF6 (1-139)-F	CGCGAGATCCTATCACTATAAAGTCATACACGTGTCTATTAGATCGT
TRAF6 (1-139)-R	TAGTGATAGGATCTCGCGCTTGGCAAAGTTATC
TRAF6 (140-597)-F	CGGATCCATGCTAACTGTTCGCTGTCCGAACTC
TRAF6 (140-597)-R	ACAGTTAGCATGGATCCGAGCTCGGTACCAAGC
TRAF6 (280-597)-F	GATCCATGATGCGCTACATGGCGGAGTTCCTGC
TRAF6 (280-597)-R	TGTAGCGCATCATGGATCCGAGCTCGGTACCAA
TRAF6 (400-597)-F	GATCCATGCACGGTATCTTCATCTGGCGCCTCA

TRAF6 (400-597)-R	AGATACCGTGCATGGATCCGAGCTCGGTACCAA
TRAF6△Ring-F	CTAGCTAGCAATGAAATGCTGTCAGAAGAG
TRAF6△Ring-R	CTAGCTAGCCTCGTATTTGCTCTCTAGGG
TRAF6△ZF-F	CTAGCTAGCATGCAGGAGTTCACTCAGATG
TRAF6△ZF-R	CTAGCTAGCGAGCTCTCTCTCCTCGTAA
TRAF6△CC-F	CTAGCTAGCGGTATCTTCATCTGGCGCCT
TRAF6△CC-R	CTAGCTAGCCTCCTCGTTAGACTGACACGG
TRAF6△TRAF-C-F	CTAGCTAGCAGTCAGAGAGCCTTTGTTAA
TRAF6△TRAF-C-R	CTAGCTAGCGATGAAGATACCGTGACACT
TRAF6-GFP-F	CCGGAATTCATGGCTTGCATTGACAGC
TRAF6-GFP-R	CGCGGATCCGCTCTGGTGTCCATCAGGTAAA
calpain2a-mCherry-F	CCGGAATTCCTGGATCTTCTTCGTCG
calpain2a-mCherry-R	CGCGGATCCTATTGGCTTCACAGGCT
calpain2a-C105A-F	GGTGACGCATGGCTGTTGGCGGCCATCGCCTC
calpain2a-C105A-R	AACAGCCATGCGTCACCCAGAGCTCCCTGGCA
calpain2a-H262A-F	TAAAGGCGCAGCCTACTCGCTGACAGGCGCCG
calpain2a-H262A-R	AGTAGGCTGCGCCTTTAACCAGCTTCTGACGG
calpain2a-N286A-F	TGGTGAGGATGAGGGCACCGTGGGGTCAGGTGGAG
calpain2a-N286A-R	TGCCCTCATCCTCACCAGCTTCTCCTGTCGAC
calpain2a-△CysPc-F	ATTCGGCCTGACTCCTGACGCCATCGAAGATGA
calpain2a-△CysPc-R	TCAGGAGTCAGGCCGAATCCAGCTGCCAGGGCC

Supplementary Table 2: List of primers for qRT-PCR analysis and in this study.

Primer name	Sequences (5'-3')
IL-8-RT-F	AGCAGCAGAGTCTTCGT
IL-8-RT-R	TCTTCGCAGTGGGAGTT
IL-6-RT-F	GACTGCCCTTCCAACTAC
IL-6-RT-R	CAGATTGTCCCGCTCC
IL-1β-RT-F	CATAAGGATGGGGACAACGAG
IL-1β-RT-R	TAGGGGACGGACACAAGGGTA
calpain2a-RT-F	CCGCTCCGCAGGAAAACT
calpain2a-RT-R	GGCTCCGCCGAGGATAAA
Viperin-RT-F	ACCCGTCCAAGTCCATAC
Viperin-RT-R	TCATGTCAGCTTTGCTCC
MX1-RT-F	GCTGCTTGTTTACTCCCA
MX1-RT-R	ACCTGCATCATCTCCCTC
ISG15-RT-F	TGAACGGACACAAGACGC
ISG15-RT-R	TGAGGAATACCTGCATGG
SCRV-P-RT-F	CTCCTTCTGCGGATGCTA
SCRV-P-RT-R	TCGGGATTTGCTCTACCAG
5.8S rRNA-RT-F	AACTCTTAGCGGTGGATCA
5.8S rRNA-RT-R	GTTTTTTTTTTTTTTTGCCGAGTG
TNF-α-RT-F	GTTTGCTTGGTACTGGAATGG
TNF-α-RT-R	TGTGGGATGATGATCTGGTTG
β-actin-RT-F	GTGATGAAGCCCAGAGCA
β-actin-RT-R	CGACCAGAGGCATACAGG
