# Supplementary material for: The protease calpain2a limits innate immunity by targeting TRAF6 in teleost fish
Source: Commun Biol. 2023 Mar 31;6:355. doi: 10.1038/s42003-023-04711-7 (PMC10066338; doi:10.1038/s42003-023-04711-7)
Supplement: Supplementary file 2 — Description of Additional Supplementary Files [file 42003_2023_4711_MOESM2_ESM.pdf]

Description of Additional Supplementary Files

File name: Supplementary Data 1

Description: The list of mass spectrometry data and statistical source data that underline the graphs in figures.
